# A Novel Cis-Acting RNA Structural Element Embedded in the Core Coding Region of the Hepatitis C Virus Genome Directs Internal Translation Initiation of the Overlapping Core+1 ORF

**DOI:** 10.3390/ijms21186974

**Published:** 2020-09-22

**Authors:** Niki Vassilaki, Efseveia Frakolaki, Katerina I. Kalliampakou, Panagiotis Sakellariou, Ioly Kotta-Loizou, Ralf Bartenschlager, Penelope Mavromara

**Affiliations:** 1 Molecular Virology Laboratory, Hellenic Pasteur Institute (HPI), 11521 Athens, Greece; sevif@pasteur.gr (E.F.); e.kalliampakou@pasteur.gr (K.I.K.); panagiotissakellariou90@gmail.com (P.S.); iolykl@yahoo.gr (I.K.-L.); 2 Department of Infectious Diseases, Molecular Virology, University of Heidelberg, 69120 Heidelberg, Germany; Ralf.Bartenschlager@med.uni-heidelberg.de; 3 German Cancer Research Center (DKFZ), 69120 Heidelberg, Germany; 4 Laboratory of Biochemistry and Molecular Virology, Department of Molecular Biology and Genetics, Democritus University of Thrace, 68100 Thrace, Greece

**Keywords:** hepatitis C virus, core+1 ORF, RNA translation, stem-loops

## Abstract

Hepatitis C virus (HCV) genome translation is initiated via an internal ribosome entry site (IRES) embedded in the 5′-untranslated region (5′UTR). We have earlier shown that the conserved RNA stem-loops (SL) SL47 and SL87 of the HCV core-encoding region are important for viral genome translation in cell culture and in vivo. Moreover, we have reported that an open reading frame overlapping the core gene in the +1 frame (core+1 ORF) encodes alternative translation products, including a protein initiated at the internal AUG codons 85/87 of this frame (nt 597–599 and 603–605), downstream of SL87, which is designated core+1/Short (core+1/S). Here, we provide evidence for SL47 and SL87 possessing a novel cis-acting element that directs the internal translation initiation of core+1/S. Firstly, using a bicistronic dual luciferase reporter system and RNA-transfection experiments, we found that nucleotides 344–596 of the HCV genotype-1a and -2a genomes support translation initiation at the core+1 frame AUG codons 85/87, when present in the sense but not the opposite orientation. Secondly, site-directed mutagenesis combined with an analysis of ribosome–HCV RNA association elucidated that SL47 and SL87 are essential for this alternative translation mechanism. Finally, experiments using cells transfected with JFH1 replicons or infected with virus-like particles showed that core+1/S expression is independent from the 5′UTR IRES and does not utilize the polyprotein initiation codon, but it requires intact SL47 and SL87 structures. Thus, SL47 and SL87, apart from their role in viral polyprotein translation, are necessary elements for mediating the internal translation initiation of the alternative core+1/S ORF.

## 1. Introduction

Chronic hepatitis C virus (HCV) infection is a major cause of liver fibrosis, cirrhosis, and hepatocellular carcinoma [1,2,3]. About 71 million people globally are chronically infected [4]. No vaccine is available yet [5], but the recently approved antiviral drugs (direct acting antivirals or DAAs) are very effective against all HCV genotypes [6,7]. However, access to such treatment remains limited due to their high costs and insufficient public health infrastructures. HCV has a single-stranded positive-sense RNA genome flanked by conserved, untranslated regions (UTRs), which are required for translation and replication [8,9]. The 5′UTR contains an internal ribosome entry site (IRES), predicted to fold into four major complex secondary/tertiary structures (domains I–IV), that directs the expression of a polyprotein [10]. The polyprotein is cleaved by cellular and viral proteases into at least ten structural and nonstructural proteins (C, E1, E2, p7, NS2, NS3, NS4A, NS4B, NS5A, and NS5B) [8,9].

Moreover, the HCV genome possesses an alternative open reading frame (ORF) overlapping the core coding region in the +1 frame (known as core+1 ORF or ARF). This core+1 ORF encodes additional proteins [11,12], including a small protein that starts at the internal AUG codons 85/87 of this frame (nt 597–599 and 603–605), which is known as core+1/Short (core+1/S) [13,14,15,16]. Interestingly, the synthesis of core+1/S in cultured cells is independent from the synthesis of the viral polyprotein, suggesting the presence of an alternative translation initiation mechanism. Core+1/S is highly unstable, and it is clearly detectable only in a tagged form, as the expression of core protein in addition to proteasome activity downregulates its intracellular levels [15]. More recently, we presented evidence for the expression of core+1/S in the context of an HCV-2a (Japanese fulminant Hepatitis-1–JFH1) subgenomic replicon [17], predominantly during the early phase of viral replication, and confirmed the expression of a longer core+1 isoform (core+1/L) [18] that has been previously identified in transfected cells [13,19].

Concerning the biological relevance of core+1 protein, specific antibodies [20,21,22,23,24,25,26,27,28,29,30,31] and T-cell responses [32,33,34] have been detected in HCV-infected individuals. Moreover, studies conducted by us revealed a high prevalence of core+1 antibodies in HCV patients with advanced cirrhosis [35] and hepatocellular carcinoma (HCC) [36], suggesting that core+1 antibodies in HCV infection may present a marker for the progression of liver disease and the development of liver cancer. Consistently, we recently showed that core+1/S isoform promotes the cell cycle in hepatoma cells and enhances liver regeneration and oncogenesis in transgenic mice [37]. However, the regulatory mechanism controlling the translation initiation of core+1/S protein remains elusive.

Interestingly, phylogenetic analysis and RNase structure mapping have identified three highly conserved RNA secondary structures within the core coding region of HCV that flank the core+1/S initiator codons, which are known as SL47, SL87, and SL248 [38,39]. We have previously shown that the integrity of the first two stem-loops SL47 (nt 388–423) and SL87 (nt 427–507) is important for the translation of the viral polyprotein but not for viral RNA stability, using the JFH1 infectious clone [40,41] in cell culture and in mice xenografted with human hepatocytes [42]. These data prompted us to investigate the possibility of the presence, in this region, of a genetic element regulating core+1/S translation, by analogy to other RNA viruses [43,44,45,46,47,48].

Here, we provide evidence that SL47 and SL87 also function as a novel cis-acting element that shares main properties of an IRES element and directs core+1/S internal translation initiation at the site of core+1 frame methionine codons 85/87.

## 2. Results

### 2.1. A Novel RNA Structural Element in the Core Coding Region Drives Internal Translation Initiation of the Core+1 ORF

**Figure 1:**
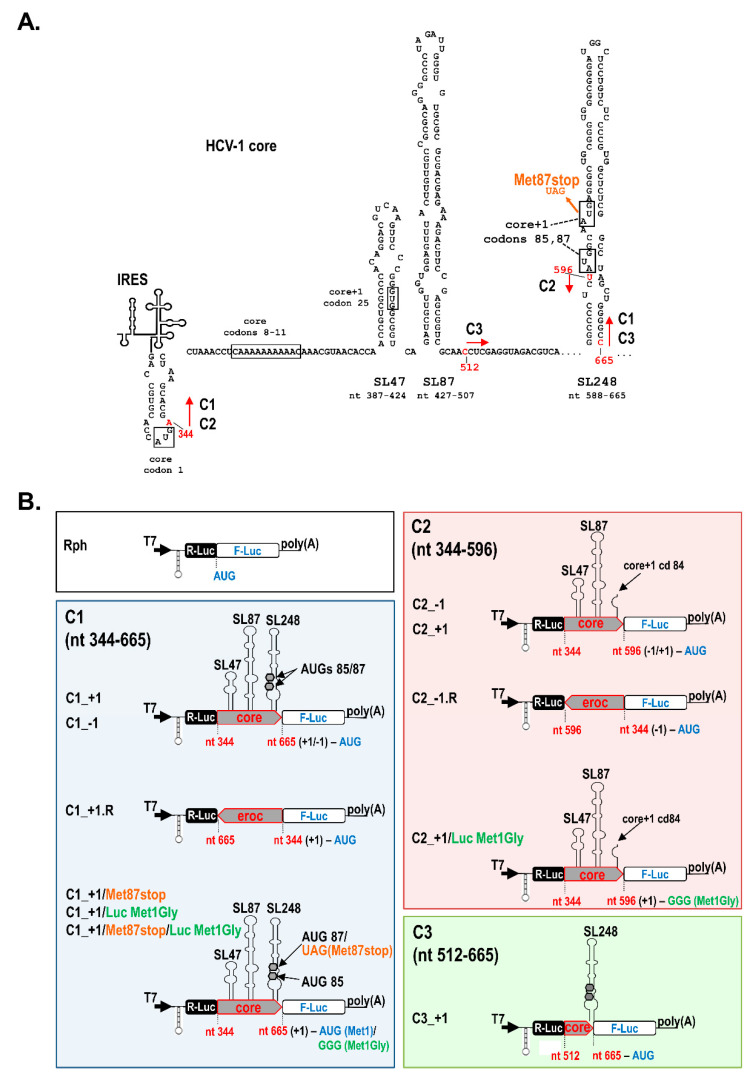
Schematic representation of the bicistronic plasmids constructed to investigate the mechanism directing core+1/Short (core+1/S) expression. (**A**) Scheme of HCV-1 (Hepatitis C virus) core coding region, including arrows indicating the start and end positions of the sequences (C1, C2, C3) inserted in the dual luciferase plasmids, relative to the predicted RNA secondary structures SL47, SL87, and SL248. (**B**) Diagram of the bicistronic constructs used. The parental vector is Renilla-photinus hairpin (Rph) (**B top left**), which has a stable hairpin (h) loop immediately upstream of the R-Luc gene and carries the Rph cassette under the control of a T7 promoter. Between the R-Luc and Firefly luciferase (F-Luc) genes, the parts of the HCV-1 core nucleotide sequence 344–665 (C1), 344–596 (C2), or 512–665 (C3) were cloned. Core sequences were inserted in the sense 5′-3′ or reverse (R) 3′-5′ orientation, and F-Luc gene was fused in the core+1 or core−1 frame. The Met87stop non-sense mutation at core+1 frame codon 87 (panels a, b for C1 construct) and Luc Met1Gly double substitution converting the F-Luc initiation codon ATG to glycine codon GGG (panel b for C1 and C2 constructs) are also illustrated.

**Figure 2:**
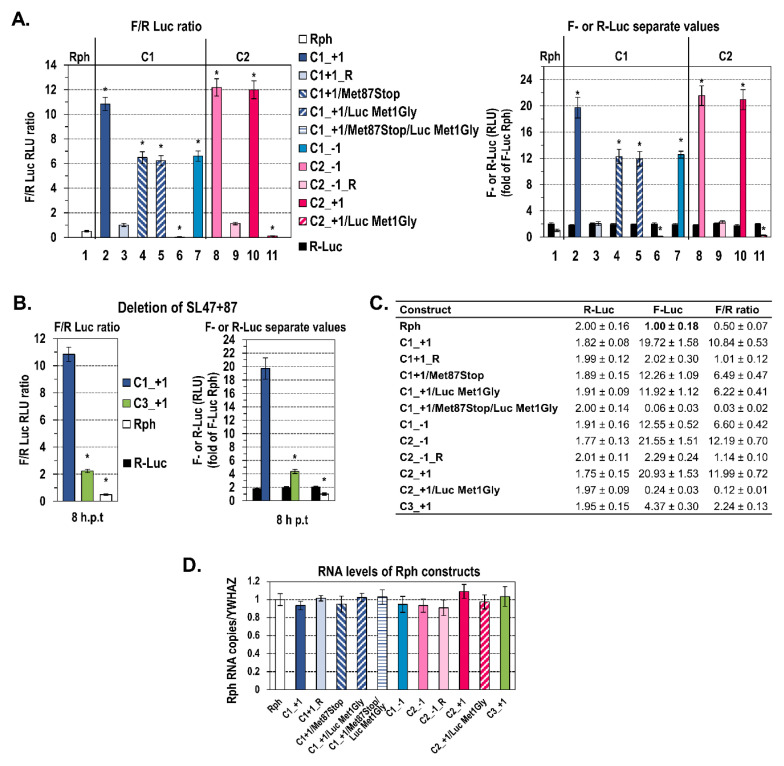
Evidence for a novel cis-acting element within the core coding sequence with capacity for internal translation initiation. (**A**) The F-Luc to R-Luc values ratio (F/R) or separate values of F-Luc and R-Luc activities detected in Huh7 cells after transfection with in vitro transcribed, capped, and polyA-tailed Rph-derived RNA containing either C1 or C2 core nucleotide sequence, in 5′-3′ or reverse (R) orientation, and the F-Luc gene fused in the core+1 or core−1 frame, in the absence or presence of the mutation Met87stop and/or substitution Luc Met1Gly. Cells were cultured for 8 h post-transfection initiation (h p.t). Values are means ± SD of four independent experiments in triplicate, expressed as relative light unit (RLU) ratio (**left**) or as separate F-Luc and R-Luc RLUs (**right**). F-Luc and R-Luc values obtained from cells transfected with the different constructs were expressed relative to the F-Luc value of the negative control empty vector Rph, which was set to one, and their ratios were calculated. Total protein amount was used for normalization. * *p* < 0.001 cells transfected with C1, C2 constructs vs. Rph empty vector, 5′-3′ constructs vs. R ones, mutated constructs vs. wild-type ones (Student’s t test). (**B**) Ratio F/R (**left**) or separate values (**right**) of F-Luc and R-Luc activities derived from Huh7 cells after transfection with in vitro transcribed, capped, and polyA-tailed C3_+1 Rph RNA as compared to the C1_+1 Rph construct. Cells were lysed 8 h p.t. Mean values were obtained from four independent experiments in triplicate and expressed as RLU ratio (**left**) or as separate F-Luc and R-Luc RLUs (**right**). F-Luc and R-Luc values were expressed relative to the F-Luc value derived from cells transfected with the negative control Rph, which was set to one, and their ratios were calculated. * *p* < 0.001, vs. C1_+1-transfected cells (Student’s t test). (**C**) Table presenting the data of panel (**A**) and panel (**B**) graphs. (**D**) RNA levels quantified 8 h p.t. of Huh7 cells with in vitro transcribed RNAs from the different Rph constructs, by RT-qPCR analysis of F-Luc gene. The expression of YWHAZ cellular gene was used for normalization. Three independent experiments were performed.

To examine whether the HCV core sequence comprising the SL47, SL87, and SL248 elements (Figure 1A) can direct internal translation initiation, we applied the previously reported dual Renilla-Firefly system Rph (Renilla-photinus hairpin, Figure 1B) [49,50,51,52]. In this bicistronic system, a DNA sequence potentially carries an IRES element when it is able to drive the efficient expression of the second cistron (Firefly luciferase, F-Luc) in an orientation-dependent manner. The Rph is an improved generation of bicistronic constructs that carries a stable hairpin loop (h) upstream of the first cistron (Renilla luciferase, R-Luc). The use of this RNA structure is a generally suggested strategy, ensuring that the translation of F-Luc is stringently controlled by the sequence inserted into the intergenic region and does not depend on the translation of the first cistron [53,54,55]. The hairpin is able to restrain the cap-dependent translation of R-Luc to minimal levels; however, these remain adequate for the normalization of transfection efficiency (Figure S1). Consequently, in the presence of the hairpin, the F-Luc/R-Luc ratio of an Rph construct carrying the HCV 5′-untranslated region (5′UTR) IRES in the intergenic region was increased up to 54-fold over background (empty vector Rph), whereas in the vector lacking the hairpin structure (Rp), the increase was only up to 10-fold (Figure S1). Furthermore, to rule out the initiation of F-Luc translation due to putative splicing or cryptic promoter activities in the inserted viral sequences [53,54,55], capped and polyA-tailed bicistronic RNAs were synthesized in vitro and transfected into Huh7 cells, without the removal of liposome–RNA complexes until cell lysis. F-Luc and R-Luc levels were measured from the Rph constructs at 8 h post-transfection initiation (h p.t) (Figure 2A,C), based on previous kinetic studies showing at this time-point the highest differences over background (Figure S2A,B). The ratios of F-Luc to R-Luc values (F/R) were also determined as shown below.

Specifically, the C1 plasmid series contains the core nucleotide sequence nt 344-665 of the HCV-1 (genotype 1a) inserted into the intercistronic region of Rph, in the 5′-3′ (sense) or 3′-5′ (reverse, R) orientation and the F-Luc gene fused in the frames core−1 (C1_−1) or core+1 (C1_+1) (Figure 1B). This sequence contains the SL47 (nt 387–424), SL87 (nt 427–507), and SL248 (nt 588–665), the initiator AUG codons 85/87 (nt 597–599 and 603–605) of core+1/S, and the initiator AUG codon of luciferase (nt 665), and it lacks the initiator AUG codon of HCV polyprotein (Figure 1). Huh7 cells were transfected with RNA derived from these vectors.

The C1_+1 construct supported efficient F-Luc activity levels that were about 20-fold those of the negative control Rph empty vector (Figure 2A—lane 2 compared to lane 1, Figure 2C). However, the C1_+1. R plasmid carrying the same DNA fragment (nt 344–665) in reverse (R) orientation failed to support significant levels of F-Luc expression (lane 3). Although there are reports for antisense sequences containing AUGs that inhibit the translation of downstream reporter genes [54], this is not the case for the reverse complement of C1 sequence, as it does not carry any upstream AUGs. Thus, these results provide the first evidence for the presence of a genetic element embedded within the HCV core coding sequences nt 344–665, directing internal translation initiation. Notably, the F-Luc levels appeared to be about 2–3-fold lower than those directed by the 5′UTR IRES in both Rph and Rp constructs (Figures S1, S2A,B).

To verify the internal translation initiation site(s) that drive the F-Luc expression, the core+1/S initiation codon 87 (nt 603–605) was converted to a stop codon (Met87stop, Figure 1). Interestingly, Met87stop failed to abolish F-Luc expression; instead, the F-Luc activity was reduced by ≈40% (Figure 2A—lane 4, Figure 2C), suggesting additional translation initiation event(s) downstream of codon 87, possibly at the start codon AUG of the F-Luc gene (nt 665). Indeed, introducing a substitution that converts the F-Luc initiator AUG to the GGG Gly codon (Luc Met1Gly, Figure 1B) impaired F-Luc translation by ≈40% (C1_+1/Luc Met1Gly, Figure 2A—lane 5, Figure 2C), whereas the double mutant C1_+1/Met87stop/Luc Met1Gly failed to support detectable levels of F-Luc expression (lane 6), suggesting that either AUG can serve as a translation initiation site.

These results were confirmed with the C1_−1 construct designed to carry F-Luc fused in the core−1 frame that is not open for translation as it contains multiple stop codons. The C1_−1 construct exhibited significant levels of F-Luc translation (Figure 2A—lane 7, Figure 2C), which were similar to those detected from the aforementioned C1_+1/Met87stop sequence (lane 4), suggesting that in both C1_−1 and C1_+1/Met87 stop constructs, F-Luc is translated exclusively from its initiator AUG.

The levels of R-Luc expression (Figure 2A right, Figure 2C), as well as the RNA abundances yielded in cells (Figure 2D) 8 h after transfection, were similar among the different Rph RNAs. Moreover, in a time-course study, the kinetics of R-Luc expression (Figure S2A right, Figure S2B) and the RNA stability (Figure S2C) did not significantly differ among the constructs. Thus, we conclude that any differences in the F-Luc activity values among the constructs can be attributed to the translational level. Collectively, these results suggest that a putative cis-acting element is embedded within the highly structured N-terminal core coding sequences nt 344–665 and mediates core+1/S translation.

### 2.2. The Presence of Intact SL47 (nt 387–424) and SL87 (nt 427–507) but Not SL248 (nt 588–665) Is Necessary for Internal Translation Initiation at Codons 85/87 of Core+1 ORF

Next, to evaluate the role of the RNA structures SL47, SL87, or SL248 in the internal translation initiation, a new series of constructs was made. Firstly, C2 plasmids carrying SL47 and SL87 but not SL248 were used (Figure 1). The C2 constructs contain the F-Luc gene either in the frame −1 (C2_−1 constructs) or +1 (C2_+1 constructs) relative to the upstream HCV-1 core nucleotide sequences 344-596 and in the sense or reverse orientation. Furthermore, the AUG of F-luc at nt 597 was placed adjacent to the core+1 frame codon 84. Since the core−1 frame contains multiple stop codons upstream to the F-luc initiator AUG, the luciferase activity from the C2_−1 construct provides a direct measure of the translation efficiency of any cis-acting element within core nt 344–596. As shown in Figure 2A,Β, the expression of F-Luc mediated by C2_−1 (lane 8) was significantly higher as compared to the background levels detected from the empty vector Rph-derived RNA (lane 1), whereas the F-Luc levels driven by the same fragment in reverse orientation (C2_−1.R) were similar to the background levels (lane 9). Similar levels of F-Luc activity to the C2_−1 construct were observed from the C2_+1, which carries the F-Luc gene fused in the core+1 frame at codon 84 (Figure 2A—lane 10, Figure 2C). When a substitution that converts the initiator AUG codon of F-Luc to GGG (Luc Met1Gly, Figure 1B) was inserted in the C2_+1 construct, F-Luc expression was completely eliminated (Figure 2A—lane 11, Figure 2C). This confirms that the AUG at nt 597 (codon 85) functions as a translation initiation codon. Moreover, the deletion of both SL47 and SL87 stem-loops from the HCV-1 C1_+1 plasmid, resulting in the mutant C3_+1 (Figure 1A, Figure 1B bottom right), abolished F-Luc expression (Figure 2B,C). Thus, core nt sequence 344–596, containing SL47 and SL87 but not SL248, can direct internal translation initiation at the AUG start codons at core+1 frame position 85/87. Notably, the levels of luciferase activity between the C1_+1 and C2_+1 constructs were equivalent (lanes 2 and 10), suggesting that SL47 and/or SL87 and not SL248 are the key elements for core+1/S translation initiation.

**Figure 3:**
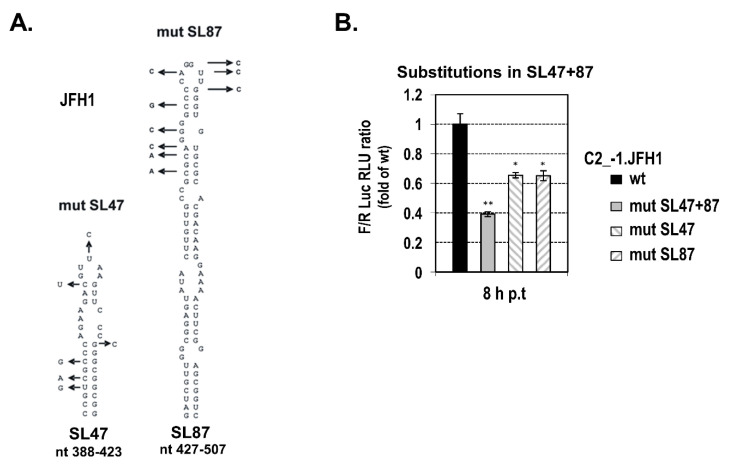
Mutation analysis of SL47 and SL87. (**A**) Schematic representation of the predicted secondary structures of SL47 and SL87 of JFH1 including substitutions disrupting base-pair interaction (indicated with arrows). The mutations do not affect the amino acid sequence of the core protein. (**B**) Ratio F/R of F-Luc to R-Luc activity derived from Huh7 cells transfected with a C2_−1.JFH1 Rph RNA, containing either the wild-type C2 core nt fragment 344–596 of JFH1, with the F-Luc gene fused in the core−1 frame (control, set to 1), or a mutated variant carrying the substitutions specified at panel a, within SL47 (C2_−1. JFH1/mut SL47), SL87 (C2_−1. JFH1/mut SL87) or both SL47 and SL87 (C2_−1. JFH1/mut SL47+87). Cells were further cultured after transfection initiation for 8 h. Values are means ± SD of four independent experiments in triplicate, * *p* < 0.01, ** *p* < 0.001, vs. control-transfected cells (Student’s t test).

Next, to confirm that SL47 and SL87 constitute integral parts of the cis-acting translational element, another series of plasmids was constructed that carries wild-type (C2_−1.JFH1) or mutated SL47 and SL87 sequences from JFH1 isolate (genotype 2a), which have been described previously [42]. The multiple substitutions introduced in SL47 (mut SL47, Figure 3A) were predicted to alter almost the complete structure, whereas the mutations within SL87 (mut SL87, Figure 3A) were predicted to affect the upper half of the stem-loop. None of them create any AUG or stop codon in frame with luciferase [42]. Similar levels of F-Luc activity were observed from C2_−1.JFH 1 that carry core nt 344–596 of JFH1 isolate into the intercistronic region of plasmid Rph as compared to the respective HCV-1 construct C2_−1 HCV-1 (Figure S2D). Similarly to the results from C3_+1 plasmid carrying the SL47 and SL87 deletion, a significant reduction of Luc activity was observed after disruption of the integrity of both stem-loops simultaneously (C2_−1.JFH1/mut SL47+87, Figure 3B). A lower defect was caused by the disruption of SL47 or SL87 separately. These data show that the integrity of core RNA SL47 and SL87 is vital for driving internal translation initiation.

**Figure 4:**
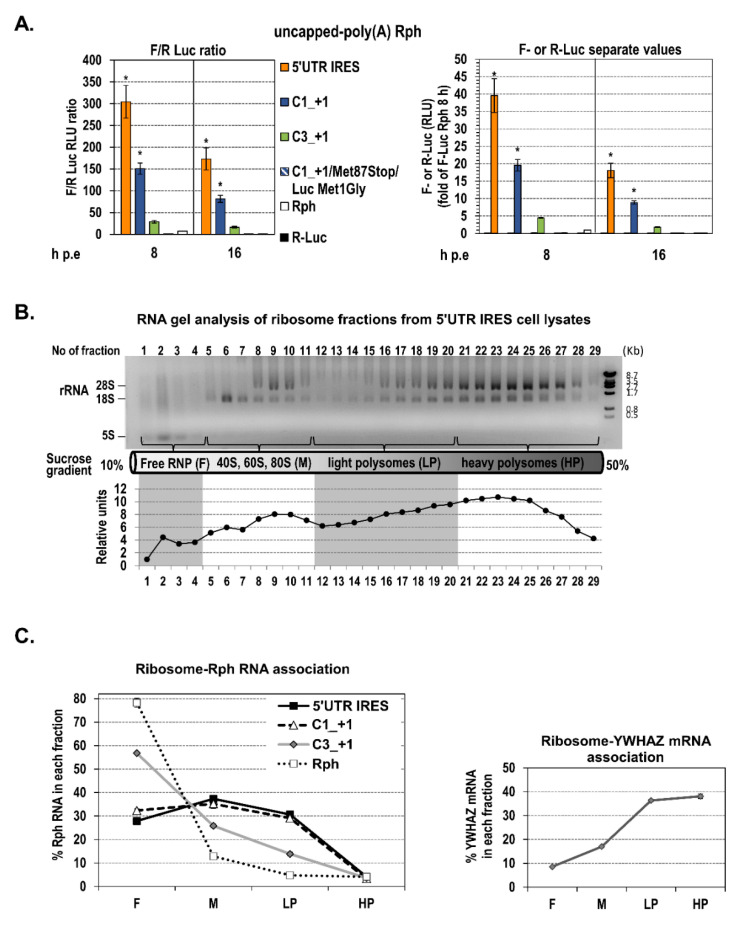
Distribution of the Rph constructs-derived RNAs in the ribosomal fractions of Huh-7 cells. (**A**) Ratio F/R (**left**) or separate values (**right**) of F-Luc and R-Luc activities determined in Huh7 cells after electroporation with in vitro transcribed, uncapped, and polyA-tailed RNA of the Rph-based 5′-untranslated region (5′UTR) internal ribosome entry site (IRES), C1_+1, C3_+1, or C1_+1/Met87stop/Luc Met1Gly constructs or the empty vector Rph, 8 and 16 h post-electroporation (p.e). Values are means ± SD of three independent experiments in triplicate and expressed as RLU ratio (**left**) or as separate F-Luc and R-Luc RLUs (**right**). F-Luc and R-Luc values for all constructs were expressed relative to the F-Luc value derived from cells transfected with the negative control Rph at 8 h p.t, which was set to one, and their ratios were calculated. * *p* < 0.001 vs. C3_+1 and vs. Rph transfected cells (Student’s t test). (**B**,**C**) The time-point of 16 h p.e. was selected to examine the loading of ribosomes on the uncapped RNAs of the Rph-based 5′UTR IRES, C1_+1, C3_+1 sequences and the respective empty vector Rph after electroporation in Huh7 cells. Cell lysates were subjected to sucrose density gradient ultracentrifugation. The distribution of ribosomal RNA along the collected fractions was determined by agarose gel electrophoresis (**B**, **top**) and quantified by Quantity One software (**B**, **bottom**). A representative gel analysis of three independent experiments is shown (of cell lysates from cells transfected with 5′UTR IRES RNA). The positions of fractions with free ribonucleoprotein complexes (F, fractions 1–4), 40S–60S–80S monosomes (M, fractions 5–11), light polysomes (LP, fractions 12–20), and heavy polysomes (HP, fractions 21–29) are depicted with arrows at the bottom of the gel, where a schematic of the 10–50% sucrose gradient is presented. Fractions from the above groups were pooled, total RNA was extracted, and the ribosome–HCV RNA association was analyzed with RT-qPCR (**C left**). The distribution of the mRNA of the housekeeping gene 14-3-3-zeta polypeptide (YWHAZ) was also analyzed, as a control (**C right**).

To confirm the capacity of SL47 and SL87 structures to mediate the core+1 frame translation, we analyzed the association of wild-type and mutated structures with polysomes, which represents an alternative approach to assess actively translating RNA population. Specifically, we compared the distribution of C1_+1 RNA with that of the respective SL47/SL87 deletion mutant (C3_+1), in the ribosomal fractions of transfected Huh-7 cells, to examine differences in their translation efficiency. 5′UTR IRES and Rph (empty vector) derived bicistronic RNAs were used as positive and negative control, respectively. In this series of experiments, we used uncapped in vitro transcripts to completely abrogate the minimal levels of cap-mediated R-Luc translation, thus restricting the binding of ribosomes exclusively to the second cistron (F-Luc). Since the Rph-derived RNAs are non-replicative, electroporation was used to optimize the transfection efficiency and subsequent ribosome association levels. Prior to the biochemical assay, the ability of the uncapped RNAs to drive F-Luc translation was tested by luciferase assay (Figure 4A). As expected, C1_+1 RNA supported efficient luciferase levels, compared to the positive control 5′UTR IRES, whereas C3_+1 (SL47/SL87 deletion mutant) yielded significantly lower levels. Rph behaved as negative control, similarly to the C1_+1/Met87stop/Luc Met1Gly construct. Then, we examined the loading of ribosomes on the bicistronic RNAs at 16 h post-electroporation (h p.e.). This time-point was selected because, in contrast to earlier time-points, at 16 h p.e., the F-Luc activity from the Rph negative control as well as R-Luc activity from all constructs were not detectable (Figure 4A). Thus, only the specific binding of ribosomes to intercistronic RNAs is expected to be detected. Moreover, at this time-point, there are still significant levels of electroporated RNA remaining in the cells, as F-Luc activity was reduced by only 2-fold compared to 8 h p.e. (Figure 4A right panel), and the RNA is still detectable and in similar quantities among the constructs (Figure S3). Ribosome–RNA complexes from transfected cells were subjected to sucrose density gradient ultracentrifugation (Figure 4B) and resulting fractions were pooled, based on the distribution of the ribosomal RNA, into four groups: the free ribonucleoprotein complexes (RNPs), 40S–60S–80S monosomes (M), light polysomes (LP), and heavy polysomes (HP). RT-qPCR analysis of these fractions showed that C1_+1 RNA is distributed in M and LP fractions at slightly lower levels as compared to 5′UTR IRES (Figure 4C). Both RNAs were almost absent from the HP fraction, which is expected as it is known that the efficiency of IRES-dependent translation is usually low compared to cap-dependent translation. On the other hand, the abundance of the C3_+1 RNA (deletion mutant), as compared to C1_+1, was significantly lower in M and LP fractions and much higher in free RNPs (*p* < 0.001). The negative control Rph empty vector-derived RNA was exclusively accumulated in free RNPs. These data are consistent with the assumption that SL47 and SL87 are important RNA elements for the translation of the HCV core+1 frame.

In total, all data from the bicistronic constructs suggest the presence of a cis-acting element embedded in SL47 and SL87 of the HCV core region that drives internal translation initiation from the core+1 frame AUG codons 85 (nt 597–599) and 87 (nt 603–605).

### 2.3. The Intact Core RNA SL47 and SL87 Structures Support Core+1/S Expression from the JFH1 Replicon

**Figure 5:**
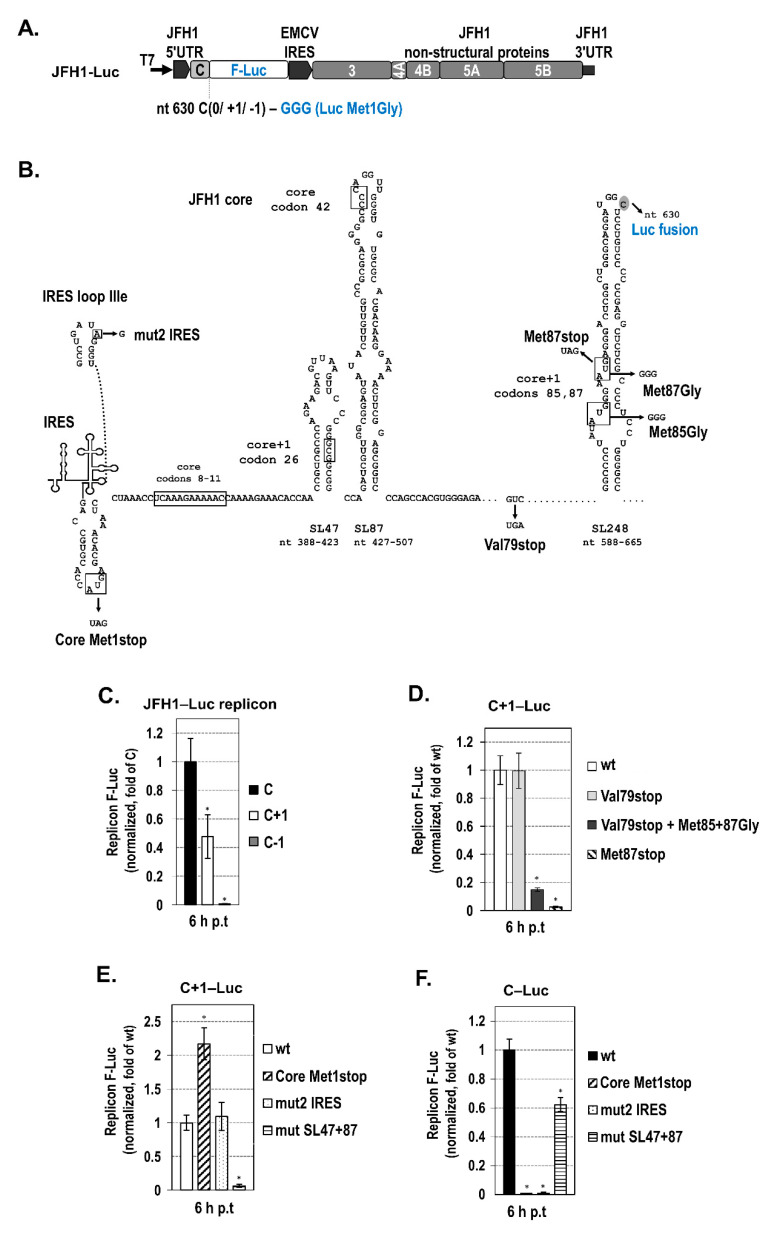
Core+1/S expression in the context of JFH1 replicon in transfected cells. (**A**) Schematic representation of the bicistronic subgenomic reporter JFH1 replicons (JFH1‒Luc) used. In the replicons, the F-Luc gene was fused to the nucleotides 1–630 of JFH1 strain, in the core (C), core+1 (C+1) or core−1 (C−1) frame. The initiator ATG of Luc has been mutated to GGG (Gly). (**B**) Schematic representation depicting the position of the mutations inserted in the JFH1‒Luc replicon, relative to the predicted core-region RNA secondary structures: non-sense substitutions at core+1 frame codon 79 abolishing all putative forms of core+1 starting before ATG initiators 85/87 and at codon 87, which in addition abrogates the expression of core+1/S, substitutions converting the core+1 initiation codons 85 and 87 to GGG (glycine), and the non-sense substitution at core codon 1, designed to abrogate polyprotein expression. In addition, the nucleotide sequence of the HCV-1 IRES loop IIIe is shown including the mut2 IRES single substitution, which is known to completely abolish IRES activity [59]. The introduced mutations are indicated with arrows that point to the substituted nucleotides. (**C**) Open reading frame overlapping the core gene in the +1 frame (Core+1 ORF) expression at the early stages of HCV replication/translation cycle. Huh7-Lunet cells were transfected with the wild-type core (C), core+1 (C+1), or core−1 (C−1) Luc replicons together with an in vitro transcribed, capped, and polyA-tailed R-Luc expressing RNA used to correct for differences in transfection efficiency. F-Luc produced by the replicons was measured at 6 h p.t. and normalized against the renilla luciferase activity derived from the co-transfected R-Luc RNA. Values for the C‒Luc replicon were set to 1 (control). Three independent experiments in triplicate were performed. (**D**) Core+1 ORF mutation analysis for elucidating the site of core+1/S translation initiation. F-Luc activity in Huh7-Lunet cells that were transfected with the wild-type C+1‒Luc replicon (control, set to 1) or a mutant carrying a non-sense mutation (stop) at core+1 frame codon 79 (Val79stop) or codon 87 (Met87stop), or Val79stop combined with glycine converting mutations at codons 85/87 (Met85+87Gly). Luciferase values were measured and normalized as described in C. Three independent experiments in triplicate were performed. (**E**,**F**) IRES mutation analysis for elucidating the mechanism of core+1/S translation. F-Luc activity in Huh7-Lunet cells that were transfected with the wild-type C+1 (**E**) or C (**F**) luciferase replicons (control, set to 1) or one of the respective mutants carrying a nucleotide substitution at core codon 1 (core Met1stop) or within IRES (mut2 IRES) or multiple substitutions disrupting RNA stem-loops SL47 and SL87 (mut SL47+87). Luciferase values were measured and normalized as described in C. Three independent experiments in triplicate were performed. Values are means ± SD, * *p* < 0.001 vs. control-transfected cells (Student’s *t* test).

Recently, we reported that core+1 ORF is expressed in the context of a bicistronic JFH1-based replicon when fused with the N-terminus of the F-Luc gene and demonstrated the production of two isoforms, core+1/L (long) and core+1/S (short), with different kinetics [18]. The core+1/S protein expression is predominant at an early point in time in the viral replication cycle, with the highest levels at 6 h post-transfection [18], which coincides with the time-point of viral RNA translation peak [56]. Based on the above data, we aimed to clarify the role of SL47 and SL87 on core+1/S expression in the context of the viral replicon [18] (Figure 5A). Luciferase tagging stabilizes the highly unstable core+1/S protein [15], and it provides a quantitative measure of the translation efficiency of core and core+1 ORFs (C‒Luc and C+1‒Luc, respectively). C−1‒Luc replicon was used as negative control, as the core−1 frame is not open for translation. In these replicons, the F-Luc initiator AUG has been mutated to GGG to exclude any translation initiation from this codon. Using this construct, we performed the following series of experiments:

First, to confirm that C+1‒Luc translation from AUG codons 85/87 is efficient at 6 h post transfection, we examined the luciferase activity of replicon C+1‒Luc, in comparison to C-Luc and to the mutant C+1‒Luc/Val79stop. The latter carries a stop codon at position 79 (nt 579–581), converting Val79 to a stop codon (Val79stop, Figure 5B). This mutation is known to abolish the production of all known core+1 isoforms initiated upstream of codons 85/87 at later times p.t. [18]. At 6 h after the transfection of Huh7 cells separately with each of the above replicon RNAs, the levels of C+1‒Luc expression from both wt (Figure 5C) and Val79Stop replicons (Figure 5D) were at ≈50% of those derived from C‒Luc, whereas only background levels were detected from C−1‒Luc (Figure 5C).

Next, to verify that the core+1 frame AUG codons 85 (nt 597–599) and 87 (nt 603–605) are active as initiators, both AUGs were converted to GGG (replicon C+1‒Luc/Met85+87Gly, Figure 5B). This conversion had a detrimental effect on F-Luc activity (Figure 5D). As expected, the Val79stop mutation in the absence of Gly substitutions had no effect of C+1-Luc expression, suggesting that core+1/S is the major form expressed at 6 h p.t. Furthermore, introducing a stop codon at position 87 (nt 603–605) totally abolished F-Luc activity (Met87stop), behaving as negative control. All the mutations described above had no effect on replicon RNA abundance (Figure S4).

Furthermore, to validate that core+1/S expression is independent from the expression of the viral polyprotein or the function of HCV IRES, we converted the core initiation codon 1 to a stop codon (Core Met1stop, Figure 5B) or inserted the previously reported A→G substitution at nt 297 of 5′UTR IRES (loop IIIe) (mut2 IRES, Figure 5B) that is known to abolish IRES-dependent translation and ribosome binding [57,58,59]. As expected, both mutations completely abolished luciferase expression in the C‒Luc replicon (Figure 5F), whereas the luciferase levels of the C+1‒Luc replicon remained unaffected (Figure 5E). No changes in the replicon RNA levels were observed (Figure S4). Together, these results support an SL47/SL87-dependent internal translation initiation mechanism, as leaky scanning or shunting require a functional IRES or initiation from an upstream initiator AUG [60], which, in the above mutated replicons, do not exist. Interestingly, the Core Met1stop mutation not only failed to abolish core+1‒Luc expression but instead increased F-Luc activity by ≈2-fold (Figure 5E). This is consistent with our previously reported data in transfected cells [14,15] and suggests that efficient translation initiation of the core+1/S protein does not require core expression. Instead, blocking the translation of the core frame has a positive effect on the translation of the core+1/S ORF. These data demonstrate that the function of 5′UTR IRES is not required for core+1/S translation in the context of virus genome and favor the presence of an alternative IRES mechanism that may drive core+1/S internal initiation.

Subsequently, to validate the importance of the integrity of core RNA SL47 and SL87 for core+1/S expression in the context of the viral sequence, the previously reported [42] multiple substitutions in SL47 and SL87 (mut SL47 and mut SL87, Figure 3A) were introduced in the C+1‒Luc JFH1-based replicon. When both SL47 and SL87 were disrupted simultaneously, only background levels of luciferase activity were detected (Figure 5E), suggesting that the core RNA SL47 and SL87 structures are vital regulatory elements for the internal translation initiation of the core+1/S protein. On the other hand, core‒Luc expression was partly impaired (Figure 5F), which is consistent with previous studies [42]. The mutations described above had no effect on replicon RNA stability/replication (Figure S4). Taken together, these results strongly suggest that SL47 and SL87 play a critical role in the expression of the core+1/S alternative reading frame protein via internal translation initiation, in a viral replicative sequence. This is consistent with the data from the Rph bicistronic system.

**Figure 6:**
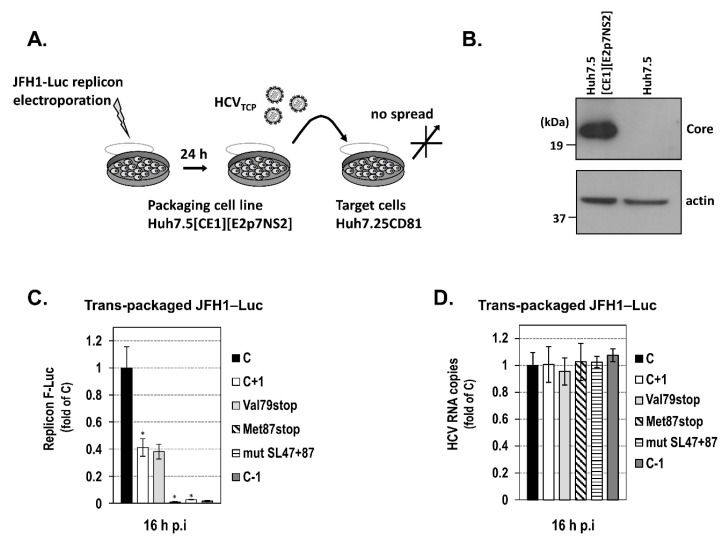
Core+1/S expression in the context of JFH1 replicon after infection with trans-complemented (TCP) viral particles. (**A**) Experimental setup to create trans-complemented HCV particles (HCV_TCP_) encapsidating subgenomic JFH1-Luc replicons in the stable cell line Huh7.5[CoreE1][E2p7NS2]. (**B**) Analysis of the CoreE1 transgene expression in electroporated with the JFH1-Luc replicon packaging cells by Western blotting using HCV core- and actin-specific antibodies. (**C**,**D**) Infection of Huh7.25-CD81 target cells by HCV_TCP_ viral particles generated upon the electroporation of Huh7.5[CoreE1][E2p7NS2] cells with one of the subgenomic JFH1-Luc replicons C, C+1, C−1, C+1/Val79stop (Val79stop), C+1/Met87stop (Met87stop), or C+1/mut SL47+87 (mut SL47+87). At 16 h post-infection (p.i.), luciferase activity levels derived from the subgenomic replicons were determined, and the intracellular HCV positive strand RNA copies were quantified by RT-qPCR. Three independent experiments were performed. Values are means ± SD, * *p* < 0.001 cells transfected with C+1 replicon vs. C, and mutated C+1 replicons with Met87stop and mutSL47+87 vs. the wild-type one (Student’s *t* test).

Finally, as the process of introducing RNAs into cells may determine their translational fate, we thought to verify our findings under conditions simulating HCV infection. Specifically, HCV trans-complemented particles (HCV_TCP_) having encapsidated the subgenomic JFH1 replicons C‒Luc, C+1‒Luc wild-type, or mutated variants thereof, or the negative control C−1‒Luc were produced in Huh7.5[CoreE1][E2p7NS2] helper cells after electroporation (Figure 6A,B) and used to infect Huh7.25-CD81 cells. A control experiment indicated that the best harvest time-point was 16 h post-infection (p.i.), which is the earliest time-point to measure detectable differences in translation levels (data not shown). Moreover, at 16 h p.i. in the trans-complementation system, the Val79stop-mutated C+1‒Luc replicon supports similar luciferase levels as the respective wild-type construct (Figure 6C), assuring that the specific time-point is appropriate for the study of core+1/S isoform, since the translation of core+1/L has not yet been initiated. Figure 6C shows significant expression from the C+1‒Luc replicon as compared to C−1‒Luc. As expected, C+1‒Luc replicons carrying the Met87stop or the stem-loop disrupting substitutions mut SL47+87 failed to support luciferase expression, inferring the implication of codons 85/87 as initiators and the role of SL47 and SL87 in core+1/S translation. Similar abundances of replicon RNAs were yielded in cells 16 h after infection with the virus-like particles (Figure 6D), reflecting a similar multiplicity of infection and replication efficiency. Thus, any differences in the luciferase activity values between the replicons can be attributed to the translational level. To conclude, previously obtained results in replicon transfected cells (Figure 5C–E) were recapitulated in the HCV_TCP_ system (Figure 6C), validating the role of SL47 and SL87 in core+1/S internal translation initiation under infection conditions.

In total, our data strongly suggest that SL47 and SL87 RNA structural elements within the HCV core coding region play an important role in directing the translation initiation of core+1 ORF at the internal core+1 frame AUG 85/87 codons, supporting the presence of an alternative IRES within the core coding sequence, as shown in other viruses [45,46,47,61,62].

## 3. Discussion

The current study provides strong evidence that the HCV core RNA stem-loops SL47 and SL87 (nucleotides 344–596) constitute a novel cis-acting element that directs the internal translation initiation at the AUG codons 85/87 (nt 597–599 and 603–605) of the alternative core ORF (core+1), which resembles the functional properties of an IRES.

We have earlier shown that the AUG codons 85/87 of the core+1 frame function as a translation initiation site of the short core+1 protein (core+1/S) in transfected cells [14,16]. Furthermore, according to recent studies [18], we have demonstrated the expression of the core+1/S ORF in the context of the bicistronic JFH1-based replicon, during the early phase of viral replication. The extensive RNA secondary structure (stem-loops SL47, 87, and 248) within the core coding region flanking the core+1/S start codons [38,39], combined with the vital role of SL47 and 87 in the translation of the viral polyprotein of the JFH1 infectious clone [42], prompted us to investigate the implication of SL47 and SL87 in core+1/S translation initiation. To this end, we followed two different approaches. First, Huh7 cells were transfected with RNAs derived from an improved generation of a bicistronic Renilla-Firefly translation system, the previously described Rph (Renilla-photinus hairpin) [49]. The use of bicistronic constructs is the most common method to determine whether a sequence is an IRES. Moreover, use of the 5′-end stem-loop, which restricts cap-dependent translation and transfection with in vitro produced RNA, instead of plasmid DNA, is a generally accepted approach to avoid artifacts [53,54,55]. With this system, we mapped the genetic element within the core region that mediates core+1/S translation. Then, the results were validated in the context of virus genome, using a JFH1-based subgenomic replicon [18] and in cells infected with trans-complemented viral particles (HCV_TCP_) carrying JFH1 replicon constructs, a system simulating native HCV entry. These experiments were combined with an extensive site directed mutagenesis approach and an analysis of ribosome-HCV RNA association.

We showed that nt 344–665 of the core-encoding region supported efficient F-luc expression when inserted only in the sense orientation in the Rph intercistronic region, suggesting for the presence of a cis-acting translation element with 30–40% efficiency as compared to the 5′UTR IRES activity. Notably, this alternative element directed translation initiation at the core+1 frame native AUG codons 85/87, as well as at the initiator AUG of the F-Luc gene fused at nt 665, which is 60 nt downstream of the proposed core+1 initiator codons. These findings suggest that the ribosome possibly enters the core region and scans downstream until an initiation codon in an appropriate context is encountered; thus, other AUG codons located close to codons 85/87 may be functional. The presence of such an AUG in the core frame has been reported in clinical isolates derived from cancer patients with high viral load of genotype 1b HCV and resistance to IFN/ribavirin [63] and has been associated with the expression of a shorter alternative form of core protein, which is known as p8 minicore [64]. On the other hand, the fusion of F-Luc at a more distant position, nt 815, of the core−1 frame (C4_−1, Figure S5) significantly reduced translation initiation compared to the respective core−1 nt 665 fusion (C1_−1). As the core−1 frame is not open for translation (it contains multiple stop codons) and, thus, in these constructs, F-Luc is translated exclusively from its initiator AUG, there are no N-terminal extensions encoded from the HCV sequence that could reduce F-Luc activity or stability.

Interestingly, mapping of the genetic element responsible for core+1/S translation initiation at codons 85/87, in both the conventional bicistronic Rph constructs and the viral subgenomic replicon system, supported the critical role of the SL47 and SL87 stem-loops. Their potency to direct translation at the position that corresponds to the internal core+1/S initiation codon 85 (nt 596) was observed when this initiator was fused either at the core+1 or core−1 frame. Deletion of both RNA stem-loops SL47 and SL87 or disruption of their integrity upon the insertion of previously reported substitutions [42] was detrimental for translation, while the separate mutation of one or the other stem-loop had a partial impact. However, in agreement to previous studies [42], the effect of these substitutions on core expression was only partial. On the other hand, translation initiation was efficient in the absence of SL248, which contains the core+1/S initiators. Thus, in the case of HCV, the RNA secondary structure that encompasses the core+1 start codons does not appear to be vital for initiation, in contrast to some other viral IRES elements (e.g., Cricket paralysis virus), where the respective RNA structure is involved in the correct positioning of the ribosomes [65]. Although a long-range kissing-loop interaction between SL87 and SL248 has been shown to affect HCV replication [66], the deletion of SL248 does not seem to alter either the 5′ UTR IRES-dependent translation, based on our previous report [42], or core+1/S translation.

The core+1/S internal translation initiation at codons 85/87 in the context of the bicistronic subgenomic JFH1‒Luc replicon and the role of SL47 and SL87 in this mechanism was verified also under conditions simulating HCV entry, upon infection of cells with HCV trans-complemented particles (HCV_TCP_) carrying the viral replicons.

Furthermore, the ability of SL47 and SL48 to mediate core+1 ORF translation was confirmed by a biochemical approach based on the distribution of RNAs in polysomes. Specifically, the distribution of the core RNA segment nt 344–665 on the different ribosome fractions was similar to the 5′UTR IRES, while the SL47/SL87 deletion mutant RNA had significantly lower abundance in the ribosomal fractions.

The presence of a functional cis-acting element supporting core+1/S internal translation initiation is further reinforced, in the context of the viral replicon, by the finding that translation initiation from core+1 codons 85/87 was independent of the 5′UTR IRES element and viral polyprotein expression. Specifically, a substitution in IRES loop IIIe that abolishes 5′UTR IRES function and ribosome binding [57,58,59] did not alter core+1-Luc expression, but it totally diminished core-Luc levels. This finding does not support core+1/S translation initiation via ribosome shunting or slippage mechanisms. Consistently, a mutation in loop IIId did not alter the translation of core+1 ORF in a conventional bicistronic plasmid, but it suppressed IRES-mediated core translation by about 60% (Figure S6), which is a reduction that is also shown in a previous study performed under similar experimental conditions [67]. Furthermore, blocking the translation initiation of the core ORF in the viral replicon not only failed to abolish core+1-Luc expression, but resulted in an increase in the luciferase activity, as previously described [14,15]. These results suggest that the synthesis of core+1/S protein is dependent on a second genetic element that may favor core+1 translation under conditions that restrict the 5-UTR IRES-driven expression of the viral polyprotein. This information, together with the finding that SL47/SL87 are solely responsible for this initiation in the context of the bicistronic Rph RNA, support that these RNA structures share the main properties of an IRES element that drives core+1 expression.

Interestingly, the SL47/SL87-driven translation is subjected to different modes of regulation as compared to the 5′UTR IRES, as its efficiency is not influenced by the viral protein NS5A (Figure S7). NS5A has been also previously shown by our group to negatively regulate the 5′UTR IRES [68], which is possibly due to a PKR/eIF-2α-dependent mechanism [69,70]. The SL47/87-dependent core+1/S expression may be regulated either through a long-range RNA–RNA interaction within the core region [71,72,73] or through interaction with core RNA binding proteins that affect IRES translation. Some examples include the nonstructural 1-associated protein 1 (NSAP1), which associates with the adenosine-rich sequence located upstream of SL47 and 87 [74], the heterogeneous ribonucleoprotein L (hnRNP L; the region spanning nt 332–402 (containing the first half of SL47) is required for its binding [75,76]), and the polypyrimidine-tract-binding protein (PTB) [77], which binds to the 3′ end of the core in addition to the 5′ and 3′UTRs facilitating genome circularization.

A hypothetical interaction between the JFH1 core/core+1 nucleotide sequence 597–604, encompassing the AUG codons 85/87, with the 18S rRNA of the 40S ribosomal subunit (Figure S8A), that would compose an IRES module regulating core+1 internal initiation [78], was not verified (Figure S8B). Moreover, although the AUGs 85/87 are well conserved among HCV isolates (Figure S9), the region complementary to the rRNA is not conserved among different genotypes and subtypes (Figure S8A), which also argues against such a contact.

Interestingly, non-primate hepaciviruses (e.g., equine, canine, Norway rat, and rodent hepaciviruses) are also predicted to have SL47/87-like RNA structures in their core region (data not shown). The presence of evolutionarily conserved RNA structural patterns in viral coding sequences has been linked to translational regulation [79], as in the case of human immunodeficiency viruses HIV-1 and HIV-2, where these patterns function as IRES elements directing the translation of shorter Gag protein isoforms at internal AUG codons [61,62].

Our results are also analogous to those reported for other RNA viruses that use IRES elements to express overlapping ORFs directing non-canonical translation initiation at internal codons. Dicistroviridae members (CrPV, IAPV, KBV, ABPV, SINV-1) use the intergenic region IRES to translate a short ORF (ORFx) that is shifted by +1 nucleotide relative to the structural polyprotein region, analogous to the HCV core+1 ORF [43,44]. In the case of Potato leafroll polerovirus (Luteoviridae), translation of an overlapping with the P1 nonstructural region ORF, known as Rap1, is controlled by an IRES located completely within the Rap1 coding region [45]. Similar to the HCV core+1 frame, the above viral alternative ORFs express short proteins. The functions of these are unknown. Moreover, in the Picornaviridae family, enteroviruses produce a short protein that is important for viral infection from an ORF partially overlapping with the viral polyprotein region at the +2 frame [46,47], and Foot-and-Mouth Disease Virus produces two alternative forms of the L protein, a peptidase, which is initiated at two in-frame AUGs [48] in an IRES-dependent mechanism.

Non-canonical translational mechanisms have been reported to regulate the expression level and/or timing of expression of various viral genes under certain conditions [80]. Thus, the alternative translation mechanism may ensure the production of core+1/S during the early stages of the viral life cycle. This could contribute to a possible role of core+1/S in controlling host antiviral innate immune response that develops very quickly after virus infection. Several DNA and RNA viruses (e.g., HIV, influenza virus, rotaviruses, noroviruses) express early proteins with such functions [80,81,82]. Consistently, preliminary data of our lab on JFH1 viruses mutated to abolish core+1/S translation without affecting the core amino acid sequence and RNA structure suggest a RIG-I-mediated decrease in viral replication caused by the absence of core+1/S in the early stages of infection.

Several positive sense RNA viruses keep surprising us with their ability to increase the coding capacity of their genomes by the use of alternative translation mechanisms based on the exploitation of secondary and higher-ordered structures as cis-regulatory elements. The new evidence on a role of SL47/87 RNA structural elements on translation of the core+1 ORF and ongoing studies on the biological function of its products would contribute to a better understanding of HCV translation and pathogenesis.

## 4. Materials and Methods

### 4.1. Cell Culture

Huh7 and replication permissive Huh7-Lunet [83] cells were cultured in high glucose (25 mM) Dulbecco’s modified minimal essential medium (Thermo Fisher Scientific, Waltham, MA, USA), supplemented with 2 mM L-glutamine, 0.1 mM non-essential amino acids, 100 U/mL penicillin, 100 µg/mL streptomycin, and 10% (*v/v*) fetal calf serum (referred to as complete DMEM). The HCV packaging cell line Huh7.5[CoreE1][E2p7NS2] (kindly provided by Thomas Pietschmann, Twincore, Hannover, Germany, [84]), which stably coexpresses the Core-E1 and E2p7NS2 regions of the Jc1 genome and supports the encapsidation of subgenomic replicons in trans-complemented HCV particles (HCV_TCP_), was cultured in complete DMEM supplemented with 5 μg/mL blasticidin. For the replication and infection-permissive Huh7.25-CD81 cells (kindly provided by Takaji Wakita, National Institute of Infectious Diseases, Tokyo, Japan, [85]), which stably express CD81 receptor, the medium was supplemented with 400 μg/mL G418.

### 4.2. Plasmid Construction and Site-Directed Mutagenesis

All nucleotide (nt) numbers refer to the JFH1 sequence (GenBank accession no. AB047639, [40]). Rph (Renilla-photinus hairpin) is a pGL3-based vector encoding a bicistronic mRNA with Renilla (R-Luc) and Photinus (Firefly, F-Luc) luciferase sequences driven by the SV40 promoter and enhancer (Figure 1B top left, kindly provided by Vincent Mauro, The Scripps Research Institute, California, USA [49]). It contains a 60-bp inverted repeat immediately upstream of the R-luc gene which, in the transcribed mRNA, forms a stable hairpin (h) loop potent to inhibit R-Luc translation. Rph derivatives constructed by PCR amplification of the core sequences or 5′UTR IRES of the HCV-1 strain (GenBank accession no. M62321) and subsequent EcoRI digestion and insertion of the amplicons in the intercistronic region between R-Luc and F-Luc are described in Table 1. C1, C2, and C3 symbolize the core sequences nt 344–665, 344–596, and 512–665, respectively (Figure 1). The core sequences have been inserted in the 5′–3′ or reverse 3′–5′ (R) orientation and with the F-Luc gene fused in the core+1 or core−1 frame (which is designated in the name of each construct). In the Rph-based constructs, the two following substitutions were introduced using the Quikchange Site-Directed Mutagenesis kit (Stratagene): Luc Met1Gly, which converts the F-Luc initiation codon to GGG (Figure 1B) and was inserted using the priming oligonucleotides 5′-GGTAAAGCCACCGGGGAAGACGCC-3′ (forward) and 5′-GGCGTCTTCCCCGGTGGCTTTACC-3′ (reverse), and Met87stop non-sense mutation, which was inserted at the core+1 frame codon 87 (nt 603–604, AUG→UAG, Figure 1) and previously described [14]. The JFH1 C2 sequence (nt 344–596) is derived from the JFH1 full-length clone [42]. The multiple substitutions mut SL47, mut SL87, and mut SL47+87 (Figure 3A), affecting the core RNA structures SL47, SL87, or both stem-loops simultaneously but not the core amino acid sequence (mut SL47: U391G, C392A, C394G, C403U, U406C, G415C; mut SL87: C455A, C457A, A458C, G460C, C463G, A467C, G469C, U470C, G472C), were introduced in the C2_−1.JFH1 Rph construct by PCR amplification of the core coding sequence of the respective mutated JFH1 full-length clone previously described in [42]. The 5′UTR IRES sequence contains the HCV-1a nt 8–406 and is derived by PCR amplification from the pHPI-1046 construct previously described in [86].

The bicistronic subgenomic replicon constructs pFK-I630-core-Luc/EI-NS3-3′-JFH1-dg (Figure 5A) that contain JFH1 5′UTR and part of the core region (nt 1–630) fused to the F-Luc gene in all three reading frames 0 (C‒Luc), +1 (C+1‒Luc), and −1 (C−1‒Luc), under the control of the T7 promoter, have been previously described [18]. In these replicons, the firefly luciferase start codon AUG has been mutated to GGG together with an introduction of a BglII site. The second cistron is expressed through the encephalomyocarditis virus IRES (EI) and encodes the NS3-to-NS5B sequence and 3′UTR of JFH1. The C+1‒Luc replicon variants containing the substitutions Val79stop (nt 579–581, GUC→UGA) or Met87Stop (nt 603–604, AUG→UAG), which give rise to non-sense mutations in the core+1 ORF (shown in Figure 5B), or Met85+87Gly converting both methionine (AUG) codons 85/87 of core+1 frame into glycine (GGG) codons, have been described previously [18]. Individual substitutions Met85Gly or Met87Gly (Table 2), Core Met1stop non-sense mutation at the HCV polyprotein initiation codon, and mut2 IRES substitution at nt 297 of the IRES loop IIIe that abolishes IRES activity (previously referred to as mut L19 [59]), were introduced in the template pFK-I630-core+1-Luc/EI-NS3-3′-JFH1-dg (C+1‒Luc), by performing overlap extension PCR. Core Met1stop and mut2 IRES mutations were also introduced in the C‒Luc replicon. The mutagenic primers used are specified in Table 2, and as outer primers, the forward JFH1 IRES-specific 5′-CTG TCT TCA CGC AGA AAG CG-3′ and the reverse F-Luc-specific 5′-CCG AAC GGA CAT TTC GAA G-3′ were used. The combined overlapping DNA products, after restriction with AgeI and XbaI, were inserted into the C+1‒Luc replicon. For the insertion of mutSL47+87 multiple substitutions in the C+1‒Luc and C‒Luc subgenomic replicons, the SexAI fragment of the replicons was replaced by the respective one derived from the mutated JFH1 full-length clone JFH1/mut SL47+87 previously described in [42]. The R-Luc expression plasmid used to correct for differences in transfection efficiency was constructed by deleting the F-Luc-containing XbaI/XbaI fragment of the pGL3-based Rp plasmid (kindly provided by Vincent Mauro, The Scripps Research Institute, La Jolla, CA, USA (39)).

### 4.3. In Vitro Transcription

Rph and Rp constructs were linearized with BamHI, extracted with phenol and chloroform, precipitated with ethanol, and dissolved in RNase-free water. For in vitro synthesis of capped and polyA-tailed Rph and Rp RNA molecules, 100 ng of linearized plasmid DNA per μL (≈2 μg) was used for in vitro transcription in mixtures containing 80 mM of HEPES (4-(2-hydroxyethyl)-1-piperazineethanesulfonic acid) pH 7.5, 12 mM MgCl_2_, 2 mM spermidine, 40 mM dithiothreitol (DTT), 2.5 mM of each deoxyribonucleoside triphosphate, 5 mM m7G(5′)ppp(5′)G (cap analog) (Ambion-Thermo Fisher Scientific, Waltham, MA, USA), 1 U of RNasin (Promega Corporation, Madison, WI, USA) per μL and 0.6 U of T7 RNA polymerase (Promega Corporation, Madison, WI, USA) per μL. After incubation for 1.5 h at 37 °C, 0.3 U of T7 RNA polymerase/μL reaction mixture was added, followed by another 1.5 h of incubation at 37 °C. Poly(A) tailing was performed after increasing the transcription reaction volume by 5 times and adding 1X E-PAP buffer, 2.5 mM MnCl_2_, 1 mM ATP, and 6 U of E. coli poly(A) polymerase I (Ambion-Thermo Fisher Scientific, Waltham, MA, USA) for 1 h of incubation at 37 °C. Transcription and poly(A) tailing reactions were terminated by the addition of 1.2 U of RNase-free DNase (Promega Corporation, Madison, WI, USA) per μg of plasmid DNA and 30 min of incubation at 37 °C. For in vitro synthesis of uncapped polyA-tailed Rph RNA molecules, the same protocol was used, but no cap analog was included. The RNA was extracted with acidic phenol and chloroform, precipitated with isopropanol, and dissolved in RNase-free water. Denaturing agarose gel electrophoresis was used to assess the integrity of RNA, and the concentration was determined by measurement of the optical density at 260 nm. HCV subgenomic replicon constructs were linearized with MluI and used for in vitro transcription as described previously [17].

### 4.4. Transfection with In Vitro Transcribed RNA

Transfections of Rph-capped polyA-tailed RNAs into Huh7 cells (0.5 μg RNA per 4 × 10^4^ cells) and transfections of in vitro transcribed HCV replicons (0.4 μg) together with a capped and polyA-tailed R-Luc expressing RNA used as a normalization control (0.1 μg, derived from the XbaI/XbaI deletion variant of Rp), into Huh7-Lunet cells (4 × 10^4^), were performed at 90% confluence using the liposome Lipofectamine 2000 (Invitrogen), as the manufacturer recommended, without removing liposome–RNA complexes until cell lysis. Transfections with Rph uncapped polyA-tailed RNAs into Huh7 cells and with HCV replicons into Huh7.5[CoreE1][E2p7NS2] packaging cells were performed by electroporation (10 μg of in vitro transcribed RNA was mixed with 400 μL of cell suspension containing 1 × 10^7^ cells/mL in the case of Huh7 and 1.5 × 10^7^ cells/mL in the case of Huh7.5 packaging cells), as described elsewhere [42].

### 4.5. Preparation of Trans-Complemented HCV Particles ([HCV_TCP_]Luc-NS3-5B) and Infection Assays

Concentrated stocks of HCV_TCP_ particles were generated as following: supernatants of Huh7.5[CoreE1][E2p7NS2] cells, 24 h after electroporation with subgenomic HCV C(0, +1, −1)‒Luc replicons, were harvested, passed through 0.45 μm pore size filters, and spun in Amicon Ultra-15 100 K centrifugal filter devices at 4000× *g* for 20 min, using a swinging bucket rotor. Huh7.25-CD81 cells were seeded at a density of 4 × 10^4^ cells per well of a 48-well plate and, 24 h later, they were inoculated with 100 μL of viral particles preparation diluted 1:2 (with complete DMEM) for 4 h. Luciferase activity was determined 16 h later, and viral RNA levels were quantified by RT-qPCR 48 h later, as described below.

### 4.6. Luciferase and Bradford Assays

Cells were lysed with Passive Lysis Buffer (Promega Corporation, Madison, WI, USA), and luciferase activity was quantified. For cells transfected with Rph constructs, F-Luc activity was measured using a Luciferase Assay System (Promega), as recommended by the manufacturer, and R-Luc activity was measured using 12 μM coelenterazine (Promega) in assay buffer (50 mM potassium phosphate, pH 7.4, 500 mM NaCl, 1 mM EDTA). For cells transfected with HCV replicons together with an R-Luc expressing RNA used as normalization control, F-Luc and R-Luc activities were determined using the Dual Luciferase Reporter Assay (Promega), according to the manufacturer’s instructions. Finally, for cells infected with HCV_TCP_ particles, F-Luc activity was measured using Luciferase Assay System. Measurements were taken in a GloMax 20/20 single tube luminometer (Promega) for 10 s. Luciferase activities were normalized to the total protein amount determined using the Bradford assay reagent (Pierce).

### 4.7. Total RNA Extraction and Quantification of Rph RNAs and Viral Replicons

Total RNA was extracted from cells using TRIzol reagent (Ambion-Thermo Fisher Scientific, Waltham, MA, USA), according to the manufacturer’s instructions. Rph RNAs and viral replicons were quantified with reverse-transcription (RT) and quantitative real-time polymerase reaction (qPCR). RT was performed using Moloney Murine Leukemia Virus (MMLV) reverse transcriptase (Promega Corporation, Madison, WI, USA), and oligo (dT) for the Rph RNAs, or the JFH1 IRES specific primer 5′-GGATTTGTGCTCATGGTGCA-3′ (reverse) for viral replicons. For qPCR, KAPA SYBR FAST qPCR Master Mix (Kapa Biosystems, Wilmington, MA, USA) was used. Rph RNAs were quantified using the F-Luc specific primers 5′-AGGTGGCTCCCGCTGAAT-3′ (forward) and 5′-CATCGTCTTTCCGTGCTCCA-3′ (reverse). Viral replicons were quantified using the JFH1 IRES specific primers 5′-GGCCTTGTGGTACTGCCTGATA-3′ (forward) and 5′-GGATTTGTGCTCATGGTGCA-3′ (reverse). The housekeeping gene YWHAZ was employed as an internal control (primers 5′-GCTGGTGATGACAAGAAAGG-3′ and 5′-GGATGTGTTGGTTGCATTTCCT-3′).

### 4.8. Gel Electrophoresis and Western Blot Analysis

Denaturing SDS-polyacrylamide gel electrophoresis and Western blotting was performed as described elsewhere [16]. Dilutions of 1:1000 for HCV core mouse monoclonal antibody (amino acids 1–120, Biogenesis, Poole, UK) and 1:6000 for pan-actin mouse monoclonal antibody (Merck-Millipore, Burlington, MA, USA) were used.

### 4.9. Polyribosome Fractionation and Quantification of Ribosome Associated RNAs

To examine the loading of ribosomes on the Rph bicistronic RNAs, polysome analysis was performed using sucrose density gradient ultracentrifugation. Huh7 cells were electroporated with in vitro transcribed uncapped polyA-tailed RNAs and seeded in 100 mm dishes. After 16 h, electroporated cells were washed twice with ice-cold PBS pH-7.4 containing 100 μg/mL cycloheximide (CXH) and lysed with 500 μL of polysomes lysis buffer containing 300 mM NaCl, 15 mM MgCl_2_, 15 mM Tris-HCl, pH 7.4, 100 μg/mL CXH, and 1% Triton-X 100. The cell lysates were collected, and nuclei were pelleted by centrifugation at 12,000× *g* for 10 min. The supernatants were layered on a linear 10–50% (w/v) sucrose gradient in polysome gradient buffer (300 mM NaCl, 15 mM MgCl_2_, 15 mM Tris-HCl, pH 7.4 and 100 μg/mL CXH) and centrifuged at 27,000 rpm for 4 h at 4 °C in a SW41 rotor (Beckman Coulter, Brea, CA, USA). Twenty-nine sucrose density gradient fractions of 400 μL were collected from the top, and the distribution of ribosomal RNA along the fractions was determined by agarose gel electrophoresis and quantified by Quantity One software (Bio-Rad, Hercules, CA, USA). The fractions were pooled as follows: samples 1–4 (free ribonucleoprotein complexes), samples 5–11 (40S, 60S and 80S monosomes), samples 12–20 (light polysomes), and samples 21–29 (heavy polysomes). Total RNA was extracted from the sucrose density fractions using acidic phenol/chloroform as described above for in vitro transcribed RNA. The distribution of the free and ribosome associated Rph RNA was determined with RT-qPCR, using F-Luc specific primers as described above.

### 4.10. Statistical Analysis

Results were subjected to statistical analysis using Student’s t-test. Statistical calculations were carried out using Excel Microsoft Office^®^ (Microsoft Corporation, Redmond, WA, USA).

### 4.11. RNA Secondary Structure Prediction

The mfold Web Server [87] was used for the RNA secondary structure predictions.

## Figures and Tables

**Table 1:** List of priming specific oligonucleotides used for Rph plasmid construction.

Primer	Sequence	Template	Rph Plasmid (HCV nt seq)
C1,C2_F C1_+1_R	5′-CCGGAATTCCGAGCACGAATCCTAAACCTC-3′ 5′-CGCCGGAATTCCGGGGCCCCAGCTAGGCCGAG-3′	HCV-1	C1_+1 (nt 344–665)
			C1_+1.R (nt 665–344)
C1,C2_F C1_−1_R	5′-CCGGAATTCCGAGCACGAATCCTAAACCTC-3′ 5΄-CGCCGGAATTCCGGCCCCAGCTAGGCCGAGAG-3΄	HCV-1	C1_−1 (nt 344–665)
C1,C2_F C2_−1_R	5′-CCGGAATTCCGAGCACGAATCCTAAACCTC-3′ 5′-CTCGAATTCCAGAGGGGCCAAGGGTACCC-3′	HCV-1	C2_−1 (nt 344–596)
			C2_−1.R (nt 596–344)
C1,C2_F C2_+1_R	5′-CCGGAATTCCGAGCACGAATCCTAAACCTC-3′ 5′-CCGGAATTCCGAGGGGCCAAGGGTACCCG-3′	HCV-1	C2_+1 C2_+1/Luc Met1Gly (nt 344–596)
C3_F C1_+1_R	5′- CGCCGGAATTCCTCGAGGTAGACGTCAGCC-3′ 5′-CGCCGGAATTCCGGGGCCCCAGCTAGGCCGAG-3′	HCV-1	C3_+1 (512–665)
C2_F_JFH1 C2_−1_R_ JFH1	5′-GCGCGCCGGAATTCCGAGCACAAATCCTAAACC TC-3′ 5′-CGCCGGAATTCCATAGGGGCCAGGGGCGACCTG-3′	JFH1	C2_−1.JFH1 (nt 344–596)
		JFH1/mut SL47+87 [42]	C2_−1.JFH1/ mut SL47+87
IRES_F IRES_R	5′-CCGGAATTCCTGATGGGGGCGACACTCCACC-3′ 5′-CCGGAATTCCGGGACGTCCTGTGGGCGGCGGT TGGTG-3′	pHPI-1046 [86]	5′UTR IRES (nt 8–406)

**Table 2:** List of priming specific oligonucleotides and plasmid DNA templates used for site-directed mutagenesis (mutated nucleotides are shown in bold).

Primer	Sequence	Template	Plasmid Constructed
Met85Gly F	5′-GGCCCCTATGGGGGAATGAGG-3′	C+1‒Luc (JFH1 replicon)	Met85Gly
Met85Gly R	5′-CCTCATTCCCCCATAGGGGCC-3′		
Met87Gly F	5′-CCTATATGGGAGGGAGGGACTCG-3′	C+1‒Luc (JFH1 replicon)	Met87Gly
Met87Gly R	5′-CGAGTCCCTCCCTCCCATATAGG-3′		
Core Met1stop F	5′-GCACCTAGAGCACAAATCCTAAAC-3′	C+1‒Luc C‒Luc (JFH1 replicon)	Core Met1stop
Core Met1stop R	5′-GATTTGTGCTCTAGGTGCACGGTCTACG-3′		
mut2 IRES F	5′-GGTACTGCCTGATGGGGCGCTTGCG-3′	C+1‒Luc C‒Luc (JFH1 replicon)	mut2 IRES
mut2 IRES R	5′-CGCAAGCGCCCCATCAGGCAGTACC-3′		

## Supplementary Materials

Supplementary materials can be found at https://www.mdpi.com/1422-0067/21/18/6974/s1.
