# Supplementary material for: A Novel Cis-Acting RNA Structural Element Embedded in the Core Coding Region of the Hepatitis C Virus Genome Directs Internal Translation Initiation of the Overlapping Core+1 ORF
Source: Int J Mol Sci. 2020 Sep 22;21(18):6974. doi: 10.3390/ijms21186974 (PMC7554737; doi:10.3390/ijms21186974)
Supplement: Supplementary file 1 [file ijms-21-06974-s001.pdf]

International Journal of *Molecular Sciences*

MDPI

Supplementary Data for

A novel cis-acting RNA structural element embedded in the core coding region of the Hepatitis C virus genome directs internal translation initiation of the overlapping core+1 ORF

Niki Vassilaki[1]*, Efseveia Frakolaki[1], Katerina I. Kalliampakou[1], Panagiotis Sakellariou[1], Ioly Kotta-Loizou[1], Ralf Bartenschlager[2,3] and Penelope Mavromara[1,4]*

[1] Molecular Virology Laboratory, Hellenic Pasteur Institute (HPI), 11521, Athens, Greece; nikiv@pasteur.gr (N.V.); sevif@pasteur.gr (E.F.); e.kalliampakou@pasteur.gr (K.I.K.); panagiotissakellariou90@gmail.com (P.S.); iolykl@yahoo.gr (I.K.-L.)

[2] Department of Infectious Diseases, Molecular Virology, University of Heidelberg, 69120, Heidelberg, Germany; Ralf.Bartenschlager@med.uni-heidelberg.de (R.B.)

[3] German Cancer Research Center (DKFZ), 69120, Heidelberg, Germany (R.B.)

[4] Laboratory of Biochemistry and Molecular Virology, Department of Molecular Biology and Genetics, Democritus University of Thrace, 68100, Thrace, Greece; pmavrom@mbg.duth.gr (P.M.)

* Correspondence: pmavrom@mbg.duth.gr (P.M.); nikiv@pasteur.gr (N.V.)

1. Supplementary Materials and Methods

1.1 Plasmid construction and site-directed mutagenesis

An Rp derivative has been constructed by PCR amplification of the 5'UTR IRES sequence containing the HCV-1a nt 8-406 from pHPI-1046 construct, previously described in [1], subsequent EcoRI-digestion and insertion of the amplicon in the intercistronic region between R-Luc and F-Luc. In the respective Rp and Rph mutated constructs del IRES, the SmaI-SmaI fragment (nt 130-316) containing the domain III of IRES has been deleted, abolishing IRES activity. The pCAT–C(0/+1/-1)–Luc bicistronic constructs pHPI-1331, -1333 and -1332 (Figure S6), have been described previously [2] and contain the chloramphenicol acetyltransferase (CAT) gene as the first cistron, followed by the 5'UTR and part of the core (C) sequence (nt 9-630) from the prototype HCV1 isolate (genotype 1a, GenBank acc. no. M62321, [3]) fused to the F-Luc gene, in the 0 (C), +1 (C+1), and -1 (C-1) frames, respectively. The C+1–Luc (pHPI-1333) variants pHPI-1380, that carries a nonsense substitution at core+1 codon 79 (Gly79stop), pHPI-1381, containing a nonsense substitution at core+1 codon 87 (Met87stop), and pHPI-1401, having a double mutation at core+1 codons 85 and 87 (Met85+87Gly), have been described previously [2]. Mut1 IRES is a substitution at nt 265-267 of IRES (loop IIId) and was introduced in the pCAT–C(0/+1)–Luc constructs, using the Quikchange™ kit (Stratagene), as manufacturer recommended (priming oligonucleotides: 5'-CCGAGTAGTGTTTTTCGCGAAAGGCC-3' and 5'-GGCCTTTCGCGAAAAACACTACTCGG-3'). Mutated nucleotides are shown in bold. For CMV-driven expression of NS5A protein in Huh7 cells, the plasmid pHPI-728 was used, containing the NS5A coding sequence (nt 6258-7601) from HCV-1a strain H77 into the pCI vector (Promega). Mut 18S is a single substitution at +4 position relative to the A of the AUG codon 85 and was created by overlapping PCR using the template pFK-I630-core+1-Luc/EI-NS3-3'-JFH1-dg (C+1–Luc) and the priming oligonucleotides mut 18S F 5'-GCCCCTATATGCGAATGAGGGACTC-3' and mut 18S R 5'-GAGTCCCTCATTCGCATATAGGGGC-3'. The PCR fragments were combined by PCR and inserted into the C+1–Luc replicon.

2. Supplementary Data

A.

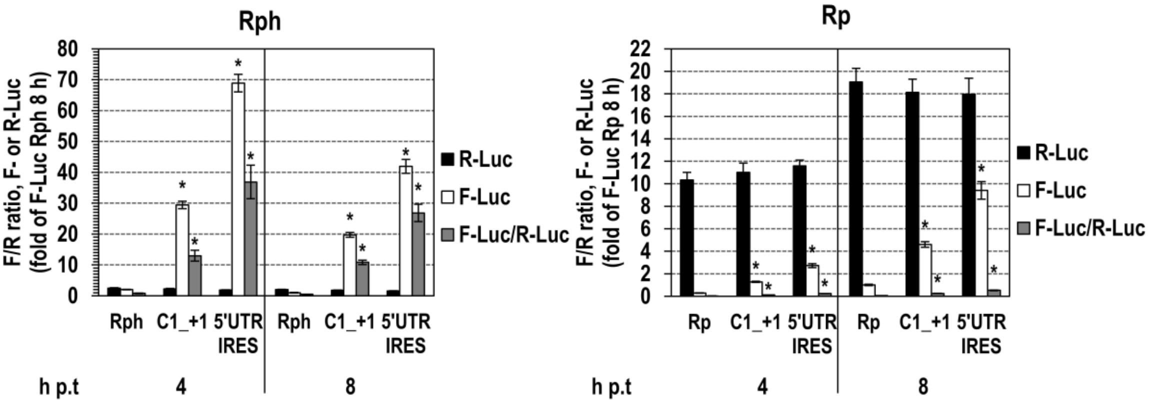

B.

h.p.t		Rph R-Luc	Rph F-Luc	Rph F/R ratio	Rp R-Luc	Rp F-Luc	Rp F/R ratio
4	Empty vector	2.47 ± 0.23	1.99 ± 0.13	0.81 ± 0.03	10.35 ± 0.67	0.29 ± 0.02	0.03 ± 0.00
	C1_+1	2.27 ± 0.21	29.43 ± 2.41	12.99 ± 0.88	11.02 ± 0.82	1.28 ± 0.57	0.11 ± 0.06
	5'UTR IRES	1.87 ± 0.19	68.91 ± 5.71	36.88 ± 2.57	11.59 ± 0.52	2.72 ± 0.18	0.23 ± 0.01
8	Empty vector	2.00 ± 0.16	**1.00 ± 0.18**	0.50 ± 0.07	19.06 ± 1.21	**1.00 ± 0.06**	0.05 ± 0.00
	C1_+1	1.82 ± 0.08	19.72 ± 1.58	10.84 ± 0.53	18.12 ± 1.18	4.60 ± 0.24	0.25 ± 0.00
	5'UTR IRES	1.56 ± 0.11	41.93 ± 4.52	26.84 ± 1.32	17.97 ± 1.41	9.42 ± 0.79	0.52 ± 0.04

C.

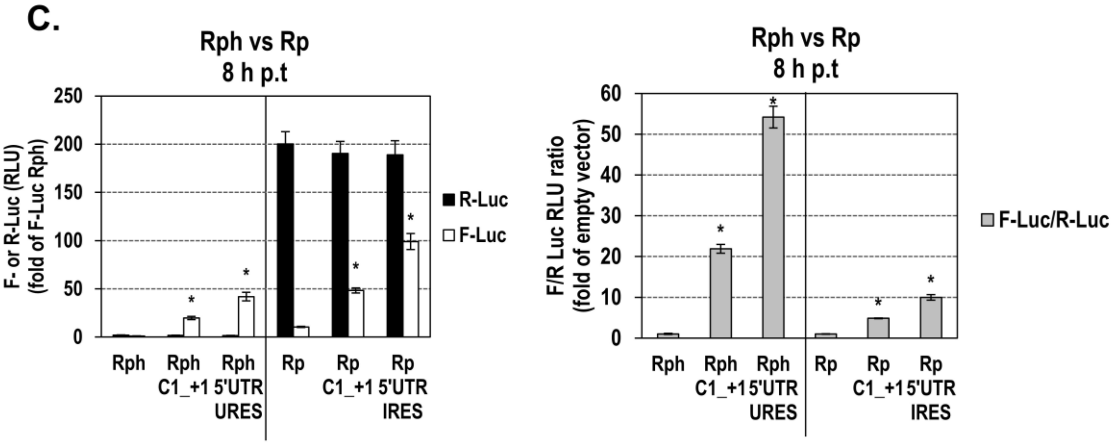

Figure S1. Comparison of internal translation initiation within the core region to the 5'UTR IRES activity in Rph and Rp constructs. (**a-c**) Ratio F/R or separate values of F-Luc and R-Luc activities derived from Huh7 cells after transfection with Rph or Rp-based RNAs carrying the C1 core nt fragment 344-665 and the F-Luc gene fused in the core+1 frame (C1_+1), or the HCV-1a 5'UTR IRES element (nt 8-406) in the intercistronic region. The empty vectors Rph or Rp RNA were used as negative controls. Cells were further cultured after transfection for the indicated hours. Values are means ± SD of three independent experiments in triplicate and expressed as RLU ratio or as separate F-Luc and R-Luc RLUs. (b) Table presenting the data of panel (a) graphs. In (a), F-Luc and R-Luc values were expressed relative to the F-Luc values for the Rph (a left) or Rp (a right) RNA at 8 h post-transfection initiation (h p.t), which were set to 1. In (c), for the comparison between Rph and Rp, F-Luc values for Rph (left) or the ratio F/R of the empty vector Rph and Rp (right) were set to 1. * p<0.001 vs Rph or Rp empty vector transfected cells (Student's t test). The presence of the hairpin increased the F/R ratio of the 5'UTR IRES construct over the background (empty vector) from 9.42-fold (Rp) to 41.93-fold (Rph), in agreement to previous data for other IRES sequences [4]. A similar increase was observed for the C1_+1 sequence, from 4.60-fold (Rp) to 19.72-fold (Rph). The highest increase versus the Rph vector was observed at 8 h p.t. (**c**)

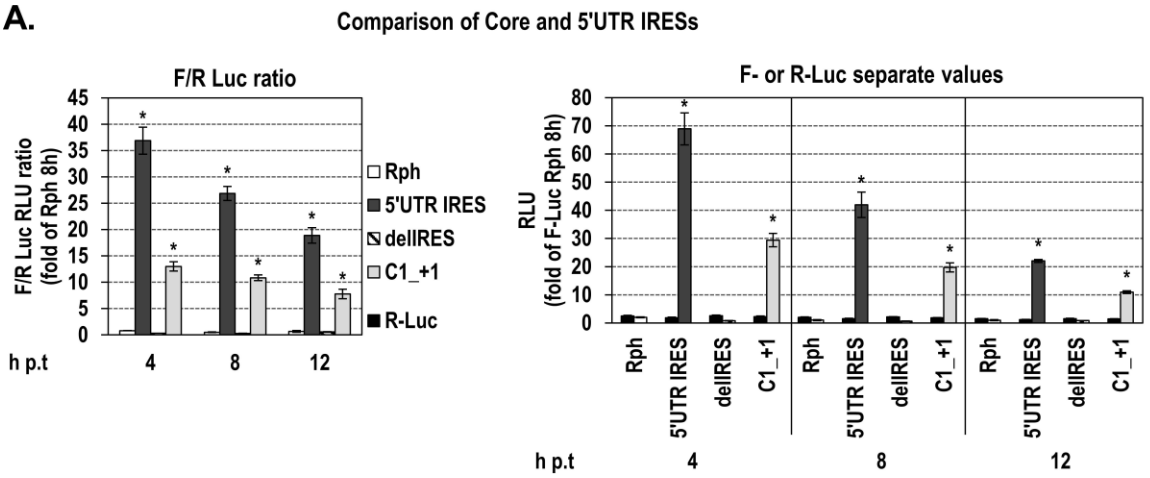

B.

h.p.t	Construct	R-Luc	F-Luc	F/R ratio
4	Rph	2.47 ± 0.23	1.99 ± 0.13	0.81 ± 0.03
	5'UTR IRES	1.87 ± 0.19	68.91 ± 5.71	36.88 ± 2.57
	delIRES	2.59 ± 0.18	0.80 ± 0.08	0.31 ± 0.01
	C1_+1	2.27 ± 0.21	29.43 ± 2.41	12.99 ± 0.88
8	Rph	2.00 ± 0.16	**1.00 ± 0.18**	0.50 ± 0.07
	5'UTR IRES	1.56 ± 0.11	41.93 ± 4.52	26.84 ± 1.32
	delIRES	2.15 ± 0.12	0.63 ± 0.05	0.29 ± 0.01
	C1_+1	1.82 ± 0.08	19.72 ± 1.58	10.84 ± 0.53
12	Rph	1.51 ± 0.09	0.98 ± 0.18	0.65 ± 0.18
	5'UTR IRES	1.18 ± 0.08	22.02 ± 0.55	18.89 ± 1.45
	delIRES	1.54 ± 0.14	0.85 ± 0.05	0.55 ± 0.05
	C1_+1	1.42 ± 0.07	10.94 ± 0.51	7.76 ± 0.92

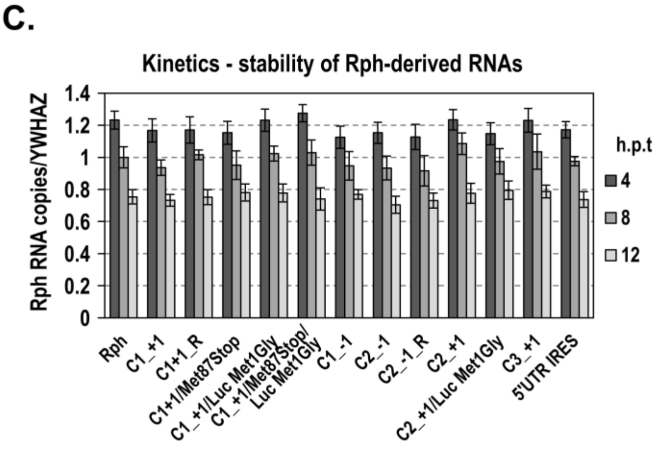

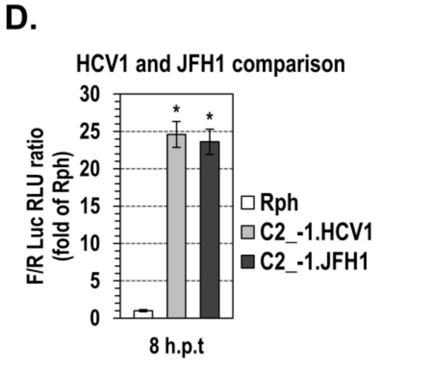

Figure S2. (a-b) Kinetics of the internal translation initiation within the core region compared to the 5'UTR IRES activity. Ratio F/R (left) or separate values (right) of F-Luc and R-Luc activities derived from Huh7 cells transfected with the Rph-based RNA C1_+1, carrying the C1 core nt fragment 344-665 and the F-Luc gene fused in the core+1 frame, compared to the respective ratio measured for the 5'UTR IRES RNA, carrying nt 8-406. The del IRES RNA, containing a deleted form of 5'UTR IRES (deletion of nt 130-316, domain III) [1], as well as the empty vector Rph RNA were used as negative controls. Cells were further cultured after transfection initiation for the indicated hours. Values are means ± SD of three independent experiments in triplicate, expressed as RLU ratio (a left) or as separate F-Luc and R-Luc RLUs (a right). (b) Table presenting the data of panel (a) graphs. F-Luc and R-Luc values for all constructs were expressed relative to the F-Luc value for the Rph RNA at 8 h p.t, which was set to 1, and their ratios were calculated. * $p<0.001$ vs Rph empty vector transfected cells (Student's t test). C1_+1 RNA supported 2-3 fold lower levels of F-Luc expression as compared to the 5'UTR IRES RNA. (c) **Kinetics of RNA levels of the Rph constructs**

5′UTR IRES, C1_+1 and empty vector Rph, 4, 8 and 12 h after transfection initiation in Huh7 cells with *in vitro* transcribed RNAs. Quantification was performed by RT-qPCR analysis of F-Luc gene using primers 5'-AGGTGGCTCCCGCTGAAT-3' and 5'-CATCGTCTTTCCGTGCTCCA-3', while the expression of YWHAZ cellular gene was used for normalization. Three independent experiments were performed. Values for the Rph RNA levels at 8 h.p.t were set to 1. (**d**) **Comparison of the internal translation initiation efficiency between HCV1 and JFH1 isolates.** Ratio of F-Luc to R-Luc activity derived from Huh7 cells after transfection with the Rph-based C2_-1.HCV1 or C2_-1.JFH1 RNA, carrying the C2 core nt fragment 344-596 of the HCV1 (1a) or JFH1 (2a) isolate, respectively, and the F-Luc gene fused in the core-1 frame (based on results of Figure 2). Cells were further cultured after transfection initiation for 8 hours. Values are means ± SD of four independent experiments in triplicate. *p<0.001 cells transfected with C2 constructs vs Rph empty vector (Student's t test).

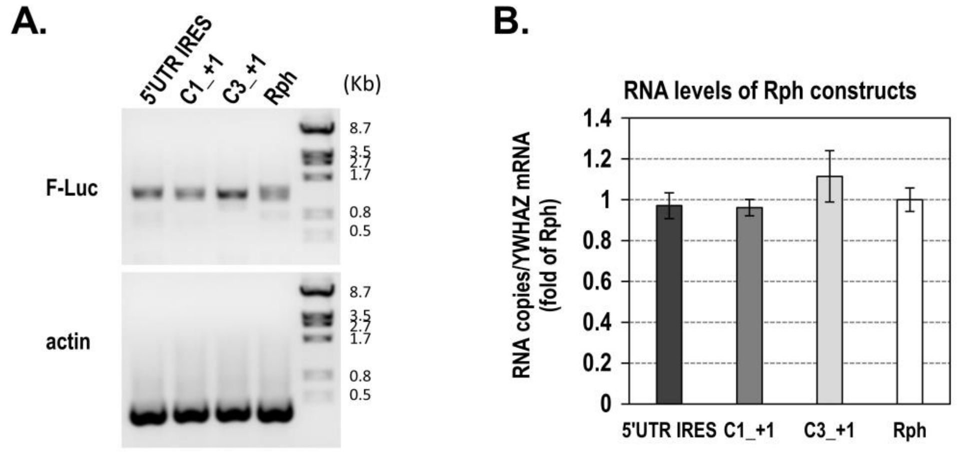

Figure S3. Levels and integrity of the uncapped Rph-based RNAs. Semi-quantitative RT-PCR (**a**) and RT-qPCR analysis (**b**) of the Rph-based RNAs 16 h after electroporation of Huh7-Lunet cells with the uncapped Rph 5'UTR IRES, C1_+1, C3_+1 sequences and the respective empty vector. RT was performed using oligo(dT) primer. (**a**) cDNAs corresponding to the entire F-Luc gene were amplified by PCR using the forward primer 5'-GGTAAAGCCACCGGGGAAGACGCC-3', specific for the 5′end of F-Luc gene and oligo(dT) as reverse primer. Actin was amplified using primers 5'-GTGTCCACCTTCCAGCAGATGT-3' (forward) and 5'-ATGCTATCACCTCCCCTGTGTG-3' (reverse) as control. PCR products were detected by agarose gel electrophoresis. A representative gel analysis of three independent experiments is shown. (**b**) RT-qPCR analysis of the F-Luc gene using primers 5'-AGGTGGCTCCCGCTGAAT-3' and 5'-CATCGTCTTTCCGTGCTCCA-3', while the expression of YWHAZ cellular gene was used for normalization. Three independent experiments were performed.

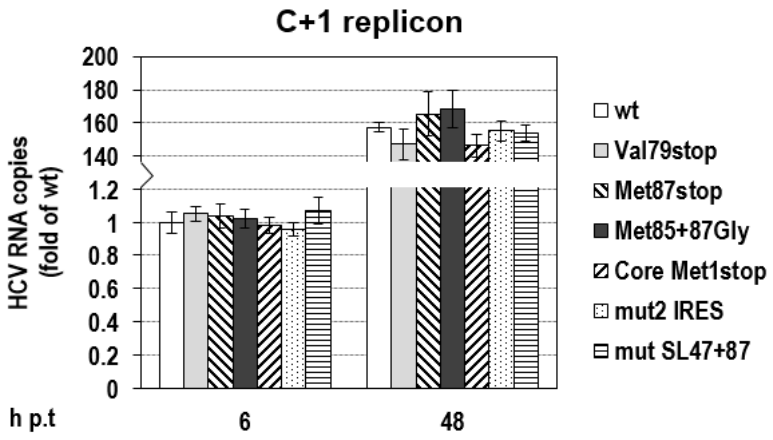

Figure S4. RT-qPCR quantification of the HCV positive strand RNA levels 6 and 48 h after transfection initiation in Huh7-Lunet cells with the wild-type C+1–Luc replicon or one of the mutants at codon 79 (Val79stop), codon 87 (Met87stop), codons 85/87 (Met85+87Gly), codon 1 (Core Met1stop), within IRES (mut2 IRES) or within stem-loops S47 and S87 (mut SL47+87), together with an *in vitro* transcribed, capped and polyA-tailed R-Luc expressing RNA used to correct for differences in transfection efficiency. Quantification was achieved by RT-qPCR analysis of the 5′UTR of the replicon, while the expression of YWHAZ cellular gene was used for normalization. Three independent experiments were performed.

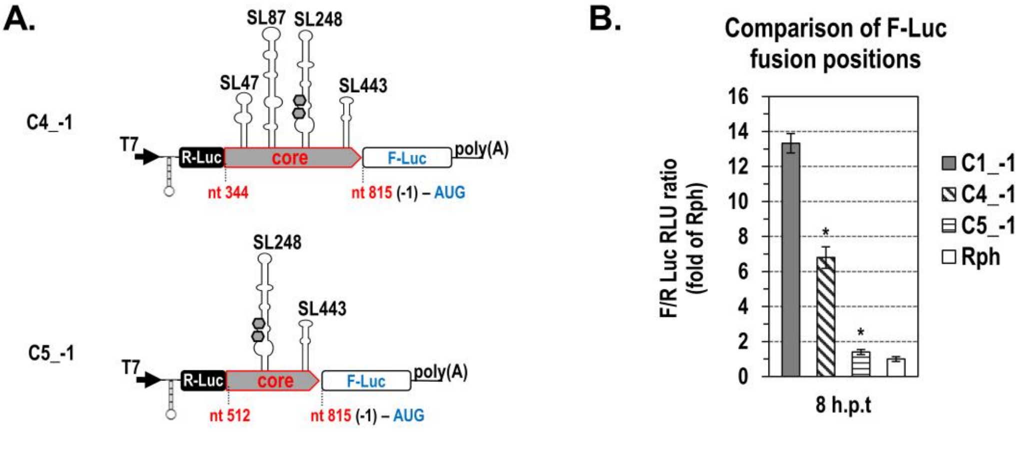

Figure S5. The core cis-acting element drives F-Luc translation with a lower efficiency when a more distant F-Luc fusion position is used. (**a**) Schematic representation of the Rph plasmids carrying, between the R-Luc and F-Luc genes, the parts of the HCV-1 core nucleotide sequence 344-815 (C4), or 512-815 (C5). The F-Luc gene was fused in the core-1 frame. The predicted RNA structures SL47, SL87, SL248 and SL443 are shown. The mfold Web Server was used for the RNA secondary structure predictions. (**b**) Ratio F/R of F-Luc to R-Luc activity detected in Huh7 cells after transfection with *in vitro* transcribed, capped and polyA-tailed Rph-derived RNA containing C1, C4 or C5 core nucleotide sequence, in 5'-3' orientation. Cells were further cultured after transfection initiation for 8 hours. Values are means ± SD of four independent experiments in triplicate and expressed as RLU ratio relative to the value derived from cells transfected with the negative control empty vector Rph. Total protein amount was used for normalization. * $p<0.001$ cells transfected with C4_-1 construct vs C1_-1 and C5_-1 construct vs C4_-1 (Student's t test).

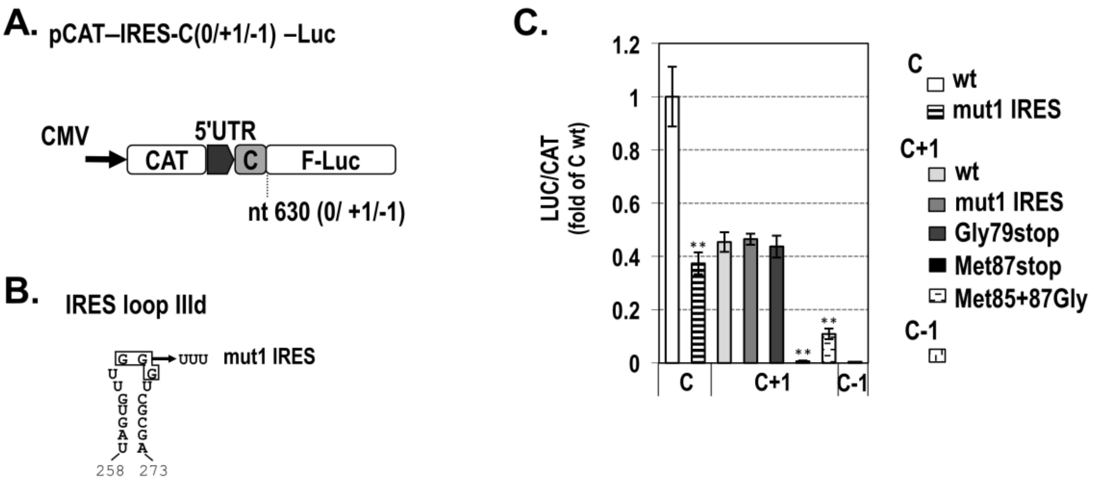

Figure S6. Core+1 ORF expression from bicistronic plasmid DNAs under the control of wild-type or a mutated HCV IRES. (**a**) Schematic representation of the pCAT–IRES-C(0,+1,-1)–Luc bicistronic constructs used for the mutation analysis. The HCV1 5'UTR IRES and part of the core sequence (nt

342-630) was inserted in the CAT-LUC intergenic region, and the F-Luc gene was fused in the -1, +1 or 0 frame of core region, under the control of CMV promoter. (**b**) Nucleotide sequence of the HCV1 5'UTR IRES loop IIIe with the mut1 IRES triple substitution (box and arrow). (**c**) Expression of the wild-type (wt) C(0,+1,-1)–Luc constructs or mutated C+1–Luc variants carrying mut1 IRES, Gly79stop, Met87stop, or Met85+87Gly, 48 h post-transfection initiation (h p.t) of *in vitro* transcribed RNA into Huh7 cells. F-Luc activity normalized to CAT quantity was determined and expressed relative to that obtained from cells transfected with the wt C-Luc construct. Bars represent mean values obtained from two independent experiments in duplicate. Error bars represent standard deviation (SD). ** p<0.01 cells transfected with mutated constructs versus wild-type ones (Student's t test).

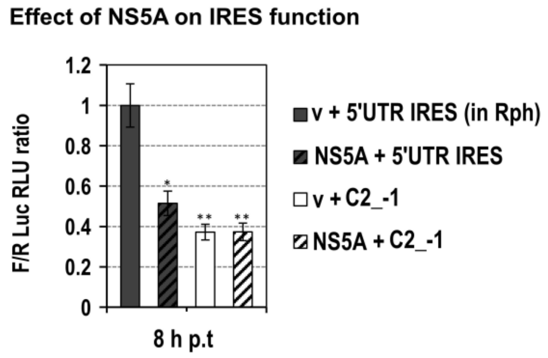

Figure S7. Differential effect of NS5A expression on the alternative IRES activity comparatively to HCV IRES. Ratio of F-Luc to R-Luc activity derived from Huh7 cells that were transfected with the CMV-NS5A plasmid DNA or the respective empty vector (v) used as a control, then (48 h p.t) transfected with the 5'UTR IRES or C2_-1 Rph-based bicitronic RNA (0.4 μg/4x10^4 cells) and further incubated for 8 hours. Mean values from three independent experiments in triplicate are expressed relative to the one determined for the cells transfected with the control empty DNA vector (v) and then the 5'UTR IRES RNA (v + 5'UTR IRES). * p< 0.05, ** p< 0.02 vs control (v + 5'UTR IRES) transfected cells, non-significant (v + C2_-1) vs (NS5A + C2_-1) (Student's t test).

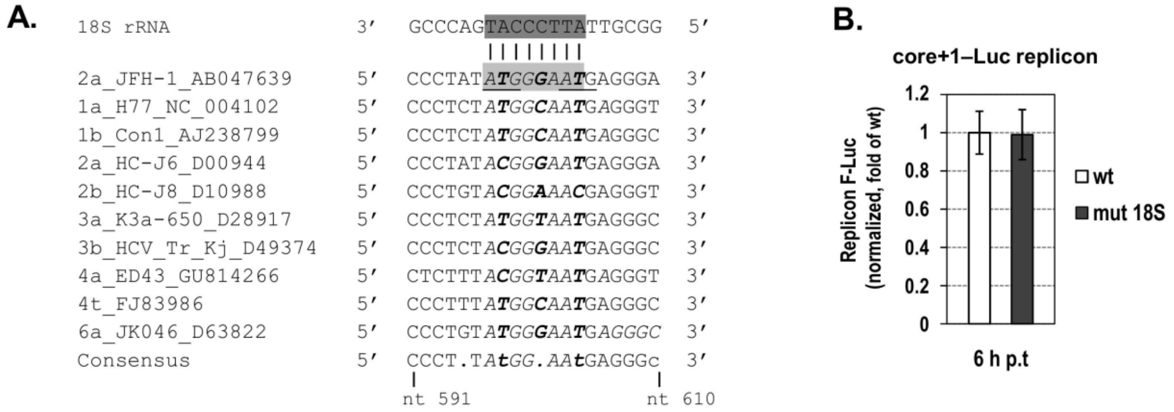

Figure S8. (**a**) At the top, a part of the human 18 S rRNA sequence in the 40S ribosomal subunit (nt 1208-1227, highlighted in gray) is given in 3′→5′ orientation. Putative base-pairing between the 18 S rRNA and the JFH1 core/core+1 nucleotide sequence 597-604 (highlighted in light gray) encompassing the AUG codons 85/87 (underlined) is indicated. Alignment of additional core nucleotide sequences from 591 to 610 of different HCV genotypes and subtypes as well as the consensus sequence for this region are included, with the nucleotides 597-604 (that in JFH1 strain are complementary with the specific 18s rRNA region) shown in italics and non-conserved positions depicted in bold. (**b**) F-Luc activity in Huh7-Lunet cells that were transfected with the wild-type C+1–Luc replicon (set to 1) or the respective mutated variant carrying a single substitution that disrupts the hypothetical complementarity of the JFH1 core+1 sequence encompassing the AUG

codons 85 and 87 with the human 18S rRNA nucleotides 1214-1221 (mut 18S). Luciferase values were measured 6 h p.t and normalized against the luciferase activity derived from the co-transfected R-Luc RNA. Three independent experiments in triplicate were performed.

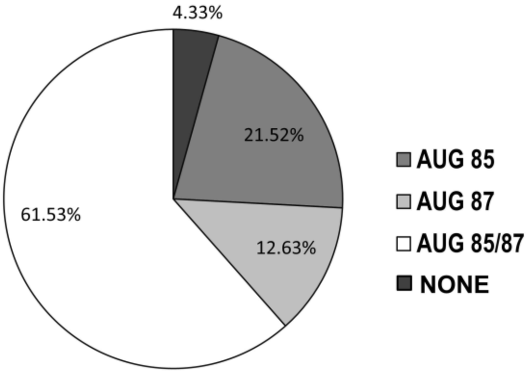

Figure S9. Conservation of the core+1 AUG codons 85 and 87 among HCV genotypes. A pie chart representation of a comparative analysis of 3374 individual HCV core sequences from the subtypes 1a-c ,1f, 2a-f, 2i, 2k, 2o, 3a-i, 3k, 4a, 4c-f, 4r, 5a, 6a-q, 6t and 7a, deposited in Los Alamos HCV sequence database, showing the percentage of sequence carrying both AUGs 85 and 87, only AUG 85, only AUG 87 or lacking both AUGs.

References

1. Kalliampakou, K.I.; Psaridi-Linardaki, L.; Mavromara, P. Mutational analysis of the apical region of domain II of the HCV IRES. *FEBS letters* **2002**, *511*, 79-84, doi:10.1016/s0014-5793(01)03300-2.
2. Vassilaki, N.; Mavromara, P. Two alternative translation mechanisms are responsible for the expression of the HCV ARFP/F/core+1 coding open reading frame. *The Journal of biological chemistry* **2003**, *278*, 40503-40513, doi:10.1074/jbc.M305504200.
3. Choo, Q.L.; Richman, K.H.; Han, J.H.; Berger, K.; Lee, C.; Dong, C.; Gallegos, C.; Coit, D.; Medina-Selby, R.; Barr, P.J., et al. Genetic organization and diversity of the hepatitis C virus. *Proc Natl Acad Sci U S A* **1991**, *88*, 2451-2455, doi:10.1073/pnas.88.6.2451.
4. Chappell, S.A.; Edelman, G.M.; Mauro, V.P. A 9-nt segment of a cellular mRNA can function as an internal ribosome entry site (IRES) and when present in linked multiple copies greatly enhances IRES activity. *Proc Natl Acad Sci U S A* **2000**, *97*, 1536-1541, doi:10.1073/pnas.97.4.1536.

© 2020 by the authors. Submitted for possible open access publication under the terms and conditions of the Creative Commons Attribution (CC BY) license (http://creativecommons.org/licenses/by/4.0/).
